# Self-harm before and during imprisonment: cohort study of males in prison linking population-based routinely collected data in Wales

**DOI:** 10.1192/bjo.2025.10898

**Published:** 2025-12-12

**Authors:** Marcos DelPozo-Banos, Mark D. Atkinson, Sze Chim Lee, Ann John

**Affiliations:** Swansea University Medical School, Faculty of Medicine, Health & Life Science, Swansea University, Swansea, UK; National Centre for Suicide Prevention and Self-Harm Research, Swansea, UK

**Keywords:** Data linkage, mental health, prisoners, routinely collected data, self-harm

## Abstract

**Background:**

Self-harm among UK prisoners has risen over the past decade.

**Aims:**

To explore self-harm risk factors and mental health conditions in prisoners, pre- and during imprisonment, compared with the general population.

**Method:**

This retrospective cohort study linked electronic health records and Ministry of Justice data for Welsh male prisoners (2019), and a comparison general population cohort. We examined imprisonment likelihood based on prior self-harm and mental health conditions using logistic regression. We also studied self-harm risk up to three years during imprisonment through Generalised Estimating Equations and time-stratified Cox regression, using a pre-imprisonment comparator (3 years before).

**Results:**

Prisoners (*N* = 6095) had higher rates of self-harm and mental health conditions pre-imprisonment compared with non-prisoners (e.g. self-harm odds ratio: 2.1 (1.9, 2.2)). Self-harm risk was 5.25–6.47 times higher in prisoners than non-prisoners, both pre- and during imprisonment. Risk was highest shortly after incarceration, then declined, becoming lower than pre-imprisonment after 7 months. While most conditions correlated with higher self-harm risk during imprisonment (e.g. drug use, hazard ratios: 1.5–3.0), some (e.g. depression and alcohol use) showed weaker links in prisoners than non-prisoners, particularly from 7 months after imprisonment. Self-harm risk was seemingly higher in prisoners on remand compared with those sentenced.

**Conclusions:**

Pre-imprisonment, self-harm in male prisoners is already high compared with the general population, potentially driving a saturation effect, where known general population risk factors have a weaker effect in prisoners. Self-harm prevention should target people in contact with criminal justice, irrespective of imprisonment. In prisons, prevention efforts deployed at inception should target those with prior self-harm, drug use, learning difficulties, bipolar disorder and those on remand.

Self-harm in people in prisons (for simplicity, from here on referred to as ‘prisoners’) is a major public health concern causing considerable morbidity.^1^ Historical examples suggest this has been the case for at least 400 years.^2^ A range of risk factors, including past and present suicidal thoughts, previous self-harm and major depression were strongly associated with self-harm in prison.^3^ These are not dissimilar to risk factors in the general population, and, similarly to other mental (e.g. psychotic illness, alcohol and drug use) and physical (e.g. hepatitis C) health conditions, they are broadly more prevalent in the prison population.^4^ However, in this population, society is more directly responsible for their safety while in prison and on discharge. Self-harm during imprisonment has been reported by 5–6% of male prisoners in the UK,^1,4^ of whom almost half had a previous history of self-harm.^5^ Previous self-harm was associated with an increased risk of suicide in prison in a systematic review and meta-analysis across 27 countries.^6^ Even after release from prison, there is a highly elevated risk of suicide among male ex-prisoners (4 times higher than in the general population) and of drug-related deaths (11 times higher).^7^

Most existing literature relies on self-reported measures which may add bias. In an Australian study, only 38% of prisoners with a pre-sentence history of self-harm (established by data-linkage) disclosed it during an interview, and the metric derived from health data was suggested to be a better predictor of subsequent self-harm than the self-reported metric.^8^ Similar strengths of using routinely collected data over self-report were reported for anxiety, depression and drug and alcohol dependency in two prisons in the north of England.^9^ Thus, examining electronic health records (EHRs) can shed light on risk factors for self-harm in prisoners and, importantly, allow comparison with non-prisoners. In a data-linkage study of non-indigenous males in Western Australia, those in prison were 22 times more likely to have a mental health service contact for self-harm in the year before imprisonment, and 10 times more likely to have any mental health service contact than non-offenders.^10^ A study based in New York City prisons linking EHRs and prison administrative data showed that serious mental illness, being under 18 and being in solitary confinement contributed significantly to self-harm in prisoners.^11^

In Welsh prisons, there was a 200% increase in recorded self-harm events between the periods 2010–2014 and 2015–2017.^12^ In 2017 alone, the number of recorded self-harm incidents increased by 16%, compared with a 10% rise in England.^12^ However, to the best of the authors’ knowledge, large-scale population-based/data-linkage studies on self-harm in prisons based on routinely collected data are scarce.^8,11^ Understanding the differences in risk factors compared with the wider population could inform comprehensive prevention approaches specifically for this group. We aimed to explore risk factors, mental health conditions and comorbidities associated with self-harm in the prison population, both before and during imprisonment, and compared with the general population. We achieved this by linking routinely collected primary care, emergency department and hospital in-patient data for the Wales population with Ministry of Justice data (MoJ) data from all Welsh prisoners in Wales in 2013–2019.

## Method

This was a retrospective electronic cohort study. The SAIL (Secure Anonymised Information Linkage) Databank Information Governance Review Panel granted ethical approval (approval number 1311). SAIL’s information governance is such that additional individual participants consent is not necessary for research underpinned by routinely collected data. Members of the public are informed of data use, and they can opt-out of their data being provided to SAIL. This study is reported in accordance with the RECORD (REporting of studies Conducted using Observational Routinely-collected Data) statements^13^ and checklist (Supplementary T1 available at https://doi.org/10.1192/bjo.2025.10898).

### Data sources

Data from the MoJ consisting of the prison population in Wales including youth offenders, during 2019, with receptions, discharges and movements information, and the Safety in Custody database with deaths, self-harm and assault events within prisons in 2013–2019 was linked within the SAIL Databank, a national repository of health and administrative data for Wales.^14,15^ A trusted third party (Digital Health and Care Wales) assigned each individual with an anonymised linkage field which allows individual-level linkage of data-sets without disclosing identifiable information. Wales has no female or category A (high security) prisons, and therefore our data was composed only of males in lower category prisons (See Supplementary Methods).

We used four data sources from SAIL in conjunction with the data provided by the MoJ. The Welsh Demographic Service Dataset (WDSD) contained demographic data of all those registered with a primary care practice (general practice, GP) in Wales, and was the spine of the population register within SAIL. The Wales Longitudinal GP (WLGP) database contained information about primary care contacts (symptoms, diagnoses, observations, test results, administrative procedures such as referrals and prescriptions) recorded as Read codes,^16^ covering around 80% of the population of Wales (periods of trustworthy WLGP data for each individual^17^). The Emergency Department Dataset (EDDS) had information about diagnoses, investigations and treatment. The Patient Episode Database for Wales recorded clinical data (diagnoses recorded by four-digit ICD-10 codes) and administrative variables of all hospital in-patient admissions in Wales. We used all records linked deterministically or probabilistically with a linkage score ≥0.9.^15^

#### Prisoner cohort

We included those in Welsh prisons during 2019 in the study if they were resident in Wales at the time of imprisonment and registered with a GP providing data to SAIL, at least, during the year before. We defined the imprisonment date for prisoners as the date they entered a Welsh prison, regardless of whether they were sentenced or on remand.

#### Non-prisoner cohort

The non-prisoner comparison group consisted of pseudo-randomly selected males born between 1930 and 2004 (exclusively matched to our prison cohort). We defined the index dates by pseudo-randomly generating dates to mirror the distributions of age at entry into the prison cohort. This two-step selection process for the non-prisoner cohort allowed us to compare cohorts with similar characteristics. We then retained those residing in Wales immediately before the index date, and with linkage to GP records and registration with a SAIL recording practice, at least, during the year before.

### Measures

Self-harm (outcome), as recorded in UK clinical records, is defined as non-fatal intentional self-poisoning or self-injury, regardless of suicidal motivation or intent, therefore including a range of behaviours such as non-suicidal self-injury and suicide attempts. This reflects that suicidal intent can be complex to assess and often ambivalent. Within the SAIL Databank, we ascertained self-harm from primary care, emergency department and hospital admission data using validated lists of codes and algorithms developed with expert clinicians (full details in Supplementary T2).^18^ The Safety in Custody database had dates and details of self-harm incidents logged by prison staff. The date of the first self-harm event following the imprisonment/index date was identified in the Safety in Custody data for prisoners, and in primary care, emergency department and hospital admission records for non-prisoners as detailed above in ‘data sources’.

We measured age (as groups <25, 25–64 and 65+ years) and deprivation level at imprisonment/index date. Deprivation level was based on the locality of residence (Lower Super Output Area (LSOA) version 2011), from the WDSD data-set. We defined five deprivation levels (from Quintile 1 – least deprived to Quintile 5 – most deprived) based on quintiles of the 2011 Welsh Index of Multiple Deprivation (WIMD) associated with LSOAs.^19^ We extracted prisoner status (remand or convicted) from MoJ data.

We generated dichotomous variables representing whether each of the following 12 conditions were recorded in prisoners’ and non-prisoners’ primary care and hospital admission data prior to imprisonment: attention-deficit hyperactivity disorder (ADHD), autism spectrum disorder (ASD), learning difficulties, conduct disorder, depression, anxiety, alcohol use, drug use, bipolar disorder, schizophrenia, other psychotic disorders and self-harm. Alcohol use, drug use and self-harm were also identified from the EDDS. Ascertainment of these events was based on validated lists of codes and algorithms developed with expert clinicians (full details in Supplementary T2).^18,20–28^ We then derived additional variables with the number of comorbidities, and the number of ‘additional comorbidities’ when considering diagnoses separately (i.e. when individual diagnoses had their own variables in the model).

### Statistical analysis

Data in SAIL is stored in a Db2-11 system, interrogated through SQL. Statistical analyses were carried out with R version 4.1.3 for Windows (R Core Team, Vienna, Austria; https://cran.rstudio.com/).

#### Comparison between prisoners and the reference population

To establish differences between prisoners and non-prisoners before imprisonment, we compared demographics, self-harm and other characteristics (see above) of these two cohorts prior to the imprisonment/index date. We ran Pearson’s chi-squared tests with Yates’ continuity correction, and univariable and multivariable stepwise logistic regression models (prisoners versus non-prisoners as the outcome variable) adjusted for individual studied conditions and number of ‘additional comorbidities’. We repeated the same stepwise analysis without including ‘additional comorbidities’. We reported effect sizes as odds ratios with 95% confidence intervals.

#### Association between pre-existing conditions and the occurrence of self-harm while imprisoned

**Fig. 1:**
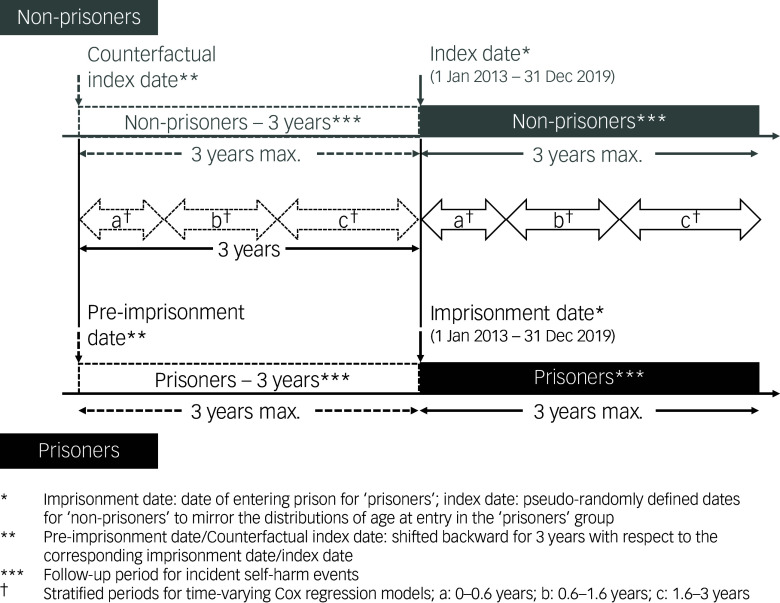
Study timeline for analysing time to incident self-harm event following imprisonment using time-stratified Cox regression. Max., maximum.

To ascertain the difference in self-harm risk between prisoners and non-prisoners both during and before imprisonment, we included a counterfactual distant index date for both cohorts 3 years before their imprisonment/index date (defined above; see Fig. 1). Only individuals with imprisonment/index dates ≥01/01/2013 were included to harmonise with the initial date of our Safety in Custody database extract. We used Generalised Estimating Equations with a log-link Poisson regression model to estimate adjusted risk ratios for the association between imprisonment and subsequent self-harm.^29^ The model included the two-way interactions of cohort (‘prisoners’, ’non-prisoners’) with time period (‘imprisonment/index date’, ‘imprisonment/index date – 3 years’) and with prior self-harm. The model was also adjusted for age groups and deprivation level. To account for the correlation among observations within individuals over time, an exchangeable working correlation structure was used, with the individual identifier as the clustering variable. The robust s.e. was estimated to account for potential misspecification of the working correlation structure. The risk ratio is >1 if subsequent self-harm risk in ‘prisoners’ is greater than in ‘non-prisoners’, =1 if equal and <1 if lower. The described interaction term between cohort and time period measures how the change in the risk ratio after the imprisonment/index date compares between prisoners and non-prisoners.

To study subsequent self-harm risk looking into the time elapsed between the imprisonment/index date and self-harm events we used Kaplan–Meier analysis and Cox regression modelling with the R ‘survival’ package version 3.8 for Windows (created by Terry M Therneau, Thomas Lumley, Atkinson Elizabeth and Crowson Cynthia; see https://therneau.r-universe.dev/survival). The Cox model also accounted for age (age group), deprivation (WIMD quintile) and pre-existing mental health conditions (12 studied conditions). We included four groups in the Cox model: (a) ‘non-prisoners’, (b) ‘prisoners’ groups with their imprisonment/index date as the start date (c) ‘non-prisoners – 3 years’ and (d) ‘prisoners – 3 years’ groups with their distant index date as the start date (see Fig. 1). Right censoring was either by death, 31/12/2019 (end date of this extract) or three years from start date (to avoid overlapping periods). We calculated time from start date to the date of incident self-harm. Independent variables were measured at or before the start date (Supplementary T2), as well as age and deprivation. We used linear combinations of coefficients to see the overall effect sizes (hazard ratios, with 95% confidence intervals) of the four groups with the R ‘biostat3’ package version 0.2.0 for Windows (created by Annika Tillander, Andreas Karlsson, Johan Zetterqvist, Peter Strom, Benedicte Delcoigne and Mark Clements; see https://cran.r-project.org/src/contrib/Archive/biostat3/). As the proportional hazard assumption was violated using Schoenfeld residuals (Supplementary T3), we carried out three time-stratified (follow-up 0 to 0.6 years – period 1, 0.6 to 1.6 years – period 2 and 1.6 to 3 years – period 3) Kaplan–Meier and Cox regression analyses (see Supplementary Methods and Supplementary F1). Hazard ratio is >1 if a variable is associated with subsequent self-harm, =1 if not associated and <1 if inversely associated. We compared hazard ratios between the four described groups to assess how the association changed between prisoners and non-prisoners, before and during imprisonment.

To study the difference in self-harm risk between sentenced prisoners and those in remand, we ran an additional three-group Kaplan–Meier analysis with (a) non-prisoners, (b) sentenced prisoners and (c) prisoners on remand. Prisoners on remand were censored at the time they are sentenced, by the end of their prison record if they were acquitted or by the other factors detailed above. We compared curves between non-prisoners, sentenced prisoners and prisoners on remand. A steeper curve indicates a faster accumulation of subsequent self-harm events. A lower end-point indicates higher subsequent self-harm risk.

## Results

### Prisoner demographics

There were 10 220 prisoners in the MoJ data-set, of which 6095 (59.6%) had the matching quality required and 4081 (39.9%) had >1 year valid GP records before the imprisonment date (Supplementary F2). At the time of imprisonment, prisoners ages ranged from 14 to 80 years, with the age distribution peaking at 22–28 years. Level of deprivation (WIMD quintile) measured at the imprisonment/index date was highly skewed, with 6% living in the least deprived and 49% in the most deprived areas (Supplementary T4). Of prisoners, 32% were on remand. Of those convicted, the most frequent sentence lengths were less than 1 year (40.7%), followed by between 1 and 2 years (18.3%).

### Pre-imprisonment self-harm and health comorbidities between prisoners and non-prisoners

History of self-harm, mental health and neurodevelopmental disorders for prisoners and non-prisoners prior to the imprisonment/index date can be seen in Table 1. All conditions were more common in prisoners than non-prisoners, with the difference always statistically significant except for ASD (Table 1 and Supplementary T4).

**Table 1:** Summary (odds ratios, with 95% CIs in parentheses) of univariable and multivariable logistic regression analyses of likelihood of being in prison

Independent variables	Unadjusted odds ratios	Adjusted odds ratios (with comorbidities)
WIMD quintile (reference: Quintile 1)		
Quintile 2	1.4 (1.2,1.7)***	1.3 (1.1, 1.6)***
Quintile 3	2.1 (1.8, 2.4)***	1.8 (1.5, 2.1)***
Quintile 4	3.4 (3.0, 3.9)***	2.5 (2.2, 2.9)***
Quintile 5 (most deprived)	6.9 (6.0, 7.8)***	4.5 (3.9, 5.1)***
Health history before imprisonment/index date		
Self-harm	11.0 (10.3, 11.8)***	4.0 (3.8, 4.4)***
Alcohol use	6.9 (6.4, 7.3)***	3.0 (2.8, 3.2)***
Drug use	18.0 (16.9, 19.2)***	8.4 (7.8, 9.0)***
Depression	5.4 (5.0, 5.7)***	1.7 (1.6, 1.8)***
Anxiety	3.9 (3.7, 4.2)***	1.3 (1.2, 1.4)***
ADHD	6.0 (5.4, 6.7)***	2.8 (2.5, 3.1)***
ASD	0.9 (0.6, 1.2) NS	0.4 (0.3, 0.6)***
Learning difficulties	1.4 (1.1, 1.7)**	0.7 (0.6, 0.9)**
Conduct disorder	5.5 (4.9, 6.3)***	2.2 (1.9, 2.5)***
Bipolar disorder	3.2 (2.4, 4.3)***	0.6 (0.5, 0.8)**
Schizophrenia	8.5 (7.5, 9.6)***	1.9 (1.7, 2.2)***
Other psychotic disorders	5.3 (4.6, 6.2)***	1.1 (0.9, 1.2) NS
Number of mental health comorbidities^a^ (reference: 0)		
1	3.3 (2.9, 3.6)***	–
2 to 5	13.0 (11.9, 14.1)***	–
More than 5	59.7 (51.8, 68.8)***	–

WIMD, Welsh Index of Multiple Deprivation; ADHD, attention-deficit hyperactivity disorder; ASD, autism spectrum disorder.

a. Self-harm included.

The dependent variable is prisoner/non-prisoner (yes/no). In column 3, the number of other comorbidities for each individual is included in each analysis.

***P-value ≤ 0.0001; **P-value ≤ 0.001; not significant (NS) *P*-value > 0.05. An odds ratio >1 indicates a variable is more common in prisoners than non-prisoners.

The number of mental health comorbidities prior to the imprisonment/index date varied considerably between prisoners and non-prisoners, with the former generally accumulating more comorbidities. Among prisoners, 17% had no comorbidities prior to imprisonment date, compared with 64% of non-prisoners. Similarly, 60% of prisoners and 17% of non-prisoners had between two and five comorbidities (Supplementary T4). Accounting for the number of comorbidities had a substantial impact on the above logistic regression results, with learning difficulties and bipolar disorders changing even effect directions – unadjusted odds ratio >1 and adjusted odds ratio <1 (Table 1).

Results from the nested logistic regressions for risk of imprisonment (Table 2) show decreasing odds ratios for history of self-harm in models accounting for other conditions and number of ‘additional comorbidities’, but always >1 and statistically significant. Similarly, gradients of odds ratios for deprivation (higher effect sizes in more deprived areas) attenuated but remained significant for all models. Drug use presented highest odds ratios across all models. Conditions with odds ratios <1 were ASD, learning difficulties, bipolar disorder and other psychotic disorders. Results excluding ‘additional comorbidities’ from the nested regressions (Supplementary T5) are similar to the main analysis.

**Table 2:** Summary (odds ratios, with 95% CIs in parentheses) of nested logistic regression analysis of health history variable ascertained before Imprisonment/index date

	Model 1		Model 2		Model 3		Model 4		Model 5		Model 6	
WIMD quintile (reference: Quintile 1)												
Quintile 2	1.3 (1.1, 1.6)***		1.3 (1.1, 1.5)**		1.3 (1.1, 1.5)**		1.3 (1.1, 1.5)**		1.3 (1.1, 1.5)**		1.3 (1.1, 1.5)**	
Quintile 3	1.8 (1.5, 2.1)***		1.7 (1.5, 1.2)***		1.6 (1.4, 1.9)***		1.6 (1.4, 1.9)***		1.6 (1.4, 1.9)***		1.6 (1.4, 1.9)***	
Quintile 4	2.5 (2.2, 2.9)***		2.4 (2.1, 2.7)***		2.2 (1.9, 2.6)***		2.2 (1.9, 2.6)***		2.2 (1.9, 2.6)***		2.2 (1.9, 2.6)***	
Quintile 5 (most deprived)	4.5 (3.9, 5.1)***		4.1 (3.6, 4.7)***		3.6 (3.2, 4.2)***		3.7 (3.2, 4.2)***		3.7 (3.2, 4.2)***		3.6 (3.2, 4.2)***	
Self-harm	–		3.6 (3.4, 3.9)***		2.1 (1.9, 2.3)***		2.1 (1.9, 2.2)***		2.1 (1.9, 2.2)***		2.1 (1.9, 2.2)***	
Alcohol use	–		–		1.5 (1.4, 1.6)***		1.5 (1.4, 1.6)***		1.5 (1.4, 1.6)***		1.5 (1.4, 1.6)***	
Drug use	–		–		6.1 (5.7, 6.7)***		6.3 (5.8, 6.8)***		6.2 (5.7, 6.8)***		6.2 (5.7, 6.7)***	
Depression	–		–		–		1.7 (1.6, 1.8)***		1.7 (1.6, 1.9)***		1.7 (1.6, 1.9)***	
Anxiety	–		–		–		1.2 (1.1, 1.3)***		1.2 (1.1, 1.3)***		1.2 (1.1, 1.3)***	
ADHD	–		–		–		–		2.4 (2.1, 2.7)***		2.4 (2.2, 2.8)***	
ASD	–		–		–		–		0.5 (0.4, 0.7)***		0.5 (0.4, 0.7)***	
Learning difficulties	–		–		–		–		0.7 (0.6, 0.9)**		0.7 (0.6, 0.9)**	
Conduct disorder	–		–		–		–		1.6 (1.4, 1.8)***		1.6 (1.4, 1.8)***	
Bipolar disorder	–		–		–		–		–		0.5 (0.4, 0.7)***	
Schizophrenia	–		–		–		–		–		1.4 (1.2, 1.6)***	
Other psychotic disorders	–		–		–		–		–		0.8 (0.7, 1.0)*	
Number of additional mental health comorbidities^a^ (reference: 0)												
1	3.0 (2.7, 3.4)***		2.9 (2.6, 3.2)***		2.0 (1.8, 2.1)***		1.7 (1.6, 1.9)***		1.2 (1.0, 1.3)‡		–	
2 to 5	11.0 (10.1, 12.0)***		7.1 (6.5, 7.8)***		2.5 (2.3, 2.7)***		1.4 (1.2, 1.6)***		0.9 (0.7, 1.1) NS		–	
More than 5	45.4 (39.3, 52.4)***		14.8 (12.0, 18.3)***		2.6 (1.3, 5.1)**		–		–		–	

WIMD, Welsh Index of Multiple Deprivation; ADHD, attention-deficit hyperactivity disorder; ASD, autism spectrum disorder.

a. The number of additional comorbidities accounting for diagnoses not explicitly included in each model. In these analyses, other comorbidities are also included, accounting for the mental health diagnoses not specifically included in each model.

The dependent variable is prisoners/non-prisoners (yes/no). ***P-value ≤0.0001; **P-value ≤0.001; *P-value ≤0.01; ‡*P*-value ≤ 0.05; not significant (NS) *P*-value >0.05. An odds ratio > 1 indicates a variable is more common in prisoners than non-prisoners.

### Risk of self-harm while in prison

Of the 4081 ‘prisoners’ at this stage of our analysis, 3780 (92.6%) were included in the ‘prisoners’ cohort and 3366 (82.5%) in the ‘prisoners – 3 years’ cohort (Fig. 1 and Supplementary F2). Of the 402 that self-harmed while in prison, 243 (60.4%) had previously presented to healthcare services with self-harm (Supplementary T6). The risk ratio of self-harm in prisoners while in prison compared with ‘non-prisoners’ was 5.80 (95% CI 5.25–6.41), and it was not statistically significantly different to the risk ratio taken 3 years earlier (imprisonment/index date – 3 year period; Fig. 1).

**Fig. 2:**
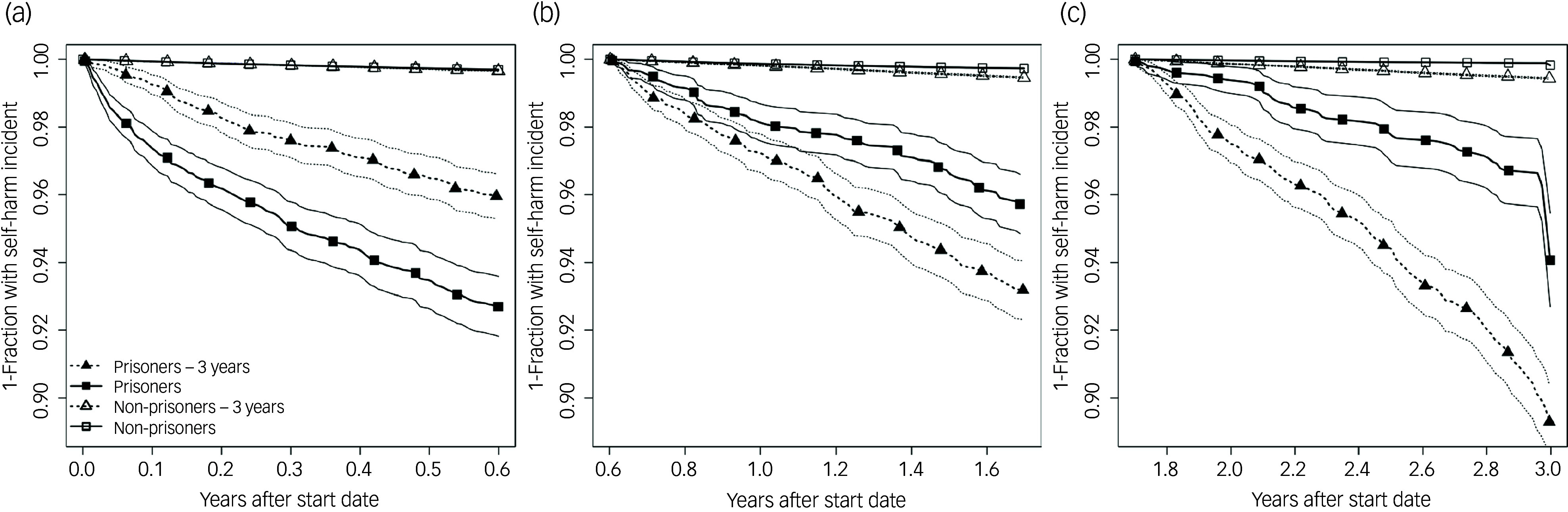
Kaplan–Meier plots for the separate time periods: (a) 0 to 0.6 years, (b) 0.6 to 1.6 years and (c) 1.6 to 3 years, showing the fraction (and 95% CIs) of prisoners and non-prisoners without self-harm incidents after their imprisonment/index dates (‘prisoners’ and ‘non-prisoners’) and after their pre-imprisonment/counterfactual index dates (‘prisoners – 3 years’ and ‘non-prisoners – 3 years’).

Time-stratified Kaplan–Meier curves for incident self-harm in ‘prisoners’ and ‘non-prisoners’ groups after imprisonment/index date are depicted in Fig. 2, and corresponding Cox regression analyses in Supplementary T7 and Supplementary F3. More self-harm occurred in prisoners within 1.6 years after the imprisonment date (periods 1 and 2) compared with non-prisoners. History of self-harm (period 1: hazard ratio 2.3; period 2: hazard ratio 1.7), ADHD (period 1: hazard ratio 1.7; period 2: hazard ratio 2.1), ASD (period 1: hazard ratio 2.4), learning difficulties (period 1: hazard ratio 1.9; period 2: hazard ratio 3.1) and drug use (period 1: hazard ratio 1.5; period 2: hazard ratio 3.0) were associated with significantly higher risk of future self-harm. Gradients of hazard risks for deprivation were observed but were not statistically significant. Individuals in the ‘non-prisoners’ group were more likely to self-harm if they had previous history of self-harm, alcohol use and depression. Within 1.6–3 years after the imprisonment date (period 3), risk of self-harm for prisoners remained higher than non-prisoners, and history of conduct disorder (hazard risk 2.7; 95% CI: 1.3–5.2) and drug use (hazard risk 2.2; 95% CI: 1.2–4.0) were risks factors for elevated risk of self-harm.

Analysis for prisoners three years before imprisonment (‘prisoners – 3 years’ group, Fig. 1) reveals higher incidence of self-harm compared with the ‘non-prisoners – 3 years’ group (Fig. 2) – a higher event than those observed in prisoners from 0.6 years after the imprisonment date (periods 2 and 3). In the ‘prisoners – 3 years’ group, only previous history of self-harm was associated with higher risk of future self-harm, while in the ‘non-prisoners – 3 years’ group, depression and drug use were also associated (Supplementary T7). Among prisoners, the link of subsequent self-harm with learning difficulties was only significant while in prison (hazard risk >1), and the link with conduct disorders, when significant, had opposite directions before and during imprisonment (hazard risk <1 in the ‘prisoners – 3 years’ group and hazard risk >1 in the ‘prisoners’ group).

Kaplan–Meier analysis including prisoners on remand shows comparable risk of self-harm up to 3 years following date of remand compared with sentenced prisoners (Supplementary F4). While those on remand showed a steeper curve than sentenced prisoners (especially after 18 months), confidence intervals were much wider due to smaller numbers, fully including that of sentenced prisoners.

## Discussion

Although the prison population has an elevated risk of self-harm, we show that this was mostly *‘brought in*’ rather than acquired during imprisonment. The risk of self-harm in prisoners was five to six times higher than in non-prisoners both 3 years prior to and during imprisonment, with no statistically significant difference between these periods. We do not rule out previously reported effects of prison environment factors such as solitary confinement, poor social support and physical or sexual victimisation while in custody.^30^ However, research including mental health factors (with lower granularity than us) highlight these as the main contributory factors to self-harm in prisoners, above environmental and relationship factors.^30,31^ All conditions and behaviours considered in this study were more likely in prisoners prior to imprisonment than in non-prisoners, even after adjusting for comorbidities – except for ASD (higher prevalence of ASD in prisoners has been reported in some forensic settings, but so has the under-diagnosis in prisons^32^), learning difficulties, bipolar disorder and other psychotic disorders. Therefore, mental health factors were already disproportionally present in prisoners pre-imprisonment, and were already associated with increased risk of self-harm.

In general, we show that prisoners were three times more likely to have a self-harm event or a mental health diagnosis than the general population, and over ten times more likely to have mental health comorbidities. Indeed, the association between mental disorders and criminal offences has been long documented and explains the over-representation of mental disorders in the prison population.^33^ This is most evident for those with a history of drug use, whose imprisonment may be related with the possession or acquisition of the drugs themselves. Such findings underscore the value of early self-harm prevention and intervention to the general population (e.g. via families and schools).

Imprisonment apparently diminished risk of self-harm in the long term. Although the risk was highest immediately after the imprisonment date, it reduced after 7 months to a level slightly higher than that of non-prisoners and lower than prisoners themselves before imprisonment. A similar observation was found in a South Carolina study,^34^ where the earlier period of imprisonment was the time of greatest risk for self-harm, and those imprisoned for shorter periods were more likely to self-harm than those with longer sentences. In a prospective cohort study in England, a reduction in ‘clinical symptoms of suicidality’ was observed even within the first 2 months of imprisonment.^35^

Our observation of a higher risk of self-harm in prisoners on remand compared with sentenced prisoners was not statistically significant. However, a similar difference has been reported elsewhere, including a lack of the above-mentioned attenuation of risk over time.^31,35^ Therefore, prisoners on remand should be considered particularly at risk of self-harm and suicidal behaviour. We suggest closely monitoring on entering prison, regardless of the receiving sentence. Overall, self-harm prevention efforts in prisons should focus on new arrivals and remain especially vigilant during the first 12 months, and throughout the remand period – until further data clarify the risk dynamics in this subgroup.

We found some evidence of a complex relationship between socioeconomic deprivation, imprisonment and self-harm. The association between high deprivation and the prison population, although strong, diminished to some extent after adjusting for previous self-harm and mental health comorbidities. Increasing deprivation increases the risk of self-harm on imprisonment relative to the risk before imprisonment. This is evident shortly after the imprisonment date (0 to 7 months) but is clearer in the medium term (7 to 19 months). Our findings are consistent with a longitudinal survey from New Zealand, showing a combination of adverse factors at individual, family, peer and school level can better explain future criminal behaviours than family socioeconomic status alone.^36^ Further research examining the effect of all these factors in combination to ascertain their relative importance is warranted, particularly including impulsivity given its suggested association with delinquency,^37^ self-harm in young people^38^ and suicide attempts in prisoners.^30^

Some well-established associations between self-harm and mental health conditions^39,40^ were weaker in prisoners than in non-prisoners, particularly prior self-harm, depression and alcohol use in relation to self-harm 1.6–3 years after the imprisonment date. For alcohol use, this is consistent with a meta-analysis of risk factors for self-harm in prison, which found no statistically significant association between the two.^3^ Interestingly, the described difference between prisoner and non-prisoner populations was also present in the 3 years before imprisonment. Therefore, the strength of these associations appears to be intrinsic to prisoners’ characteristics, and not related to the experience of imprisonment itself – notwithstanding the effect of prison environmental factors.^30^ Our results may also reflect a saturation effect by which an already elevated risk of self-harm does not increase in the same proportion when a new mental health factor presents. This saturation effect could be driven by factors common to self-harm and criminality, such as impulsivity (as described above), as well as by protective factors such as family support via visits and prohibition of alcohol or drugs during imprisonment. In the case of prior self-harm, the difference between prisoners and non-prisoners may be driven by the frequency of self-harm, particularly as the association with subsequent events weakened over time in prisoners, and models for later follow-up periods (0.6–1.6 and 1.6–3 years after imprisonment) did not capture intervening events after the imprisonment date.

We observed that the risk of self-harm for those with learning difficulties and conduct disorder was lower in prisoners 3 years before imprisonment than when in prison. This may be related to reported coping difficulties experienced by these prisoners, leading to higher levels of stress and self-harm.^41^ Other mental health conditions with a stronger effect while in prison compared to before, albeit not as marked (larger confidence intervals overlap), are drug use, anxiety and ADHD. The literature provides further evidence supporting part of this. For example, the association of ADHD with self-harming behaviour has been previously established,^42^ as well as a relatively high prevalence of ADHD in the prison system.^43^ Overall, self-harm prevention interventions in prisons should target, particularly, prisoners with learning difficulties, but also those with conduct disorders, drug use, anxiety and ADHD, as these are particularly vulnerable to the prison environment.

### Strengths and limitations

We have presented a novel analysis that compares not only prisoners with the general population, but also with themselves 3 years before imprisonment. This has been crucial in the interpretation of our results, especially when identifying the effects of imprisonment and the prison environment. Furthermore, we conducted such analysis using routinely collected population-based data for Wales (UK), linking six different data resources at person-level, including demographic, primary and secondary care, and Welsh MoJ data.

The use of linked, routinely collected clinical and administrative data reduces bias and allows larger cohorts to be studied compared with self-reported data. However, only individuals who present to healthcare services are detected and therefore we may underestimate the prevalence of the studied mental health conditions and behaviours. This is particularly true for self-harm, exemplified by the iceberg model.^44^ We combined data from primary care, emergency department and secondary (in-patient) care, and used validated diagnostic code lists in a bid to minimise such bias.

We had a relatively low linkage rate in the MoJ data (59.6%). A portion of this is as a result of prisoners who were resident in England prior to imprisonment, and therefore were not present in the Welsh Demographic Service data. Another factor may be prisoners without a permanent address or not registered with a GP, again making them unavailable for data linkage. For example, vulnerable groups of individuals such as the homeless, travellers and gypsies may be disproportionally represented in prison populations and among those not available for matching.^45^ Further investigation is needed to increase data coverage particularly to those from marginalised or underserved groups. Because of the cross-sectional nature of the MoJ data used, our non-prisoner comparison group may include ex-prisoners prior to 2019, although this is unlikely to have had a big impact in our results due to the averaging effects of our large sample. We also have limited data to quantify differences between remand and sentenced prisoners.

Self-harm in prison, and therefore in MoJ data, is ascertained differently than in population primary and secondary healthcare data. As a result, self-harm events are potentially better recorded in prison than in the community, where it highly depends on self-presentation and where it is not always identified or recorded as intentional self-harm.^44^ This is likely to have had an impact on our results and findings. However, the risk ratio of self-harm risk while in prison (based on both SAIL and MoJ data) was not statistically significantly different to the risk ratio 3 years before (based only on SAIL data). Thus, it is likely that the recording bias is smaller than anticipated. Alternatively, the incidence of self-harm in prisoners is lower while in prison compared with before, hence counteracting the recording bias. Unfortunately, we are unable to determine with certainty which of these two alternatives are driving our findings.

This study does not include important risk factors associated with criminality and self-harm, such as family and peer factors. Similarly, we did not consider childhood maltreatment in our analyses because this would have restricted the number of individuals to those with childhood data. Finally, we only have data for the prison population in 2019, so we do not know if any prisoners have had previous convictions or if they were also in prison in the 3 years before their sentence extending to 2019.

### Implications for research, policy and practice

In light of our results, early interventions for self-harm should target individuals exhibiting an inclination to unlawful behaviours, e.g. as they come in contact with the police or the justice system, and regardless of whether they are sent to prison or not. Similarly, early interventions for these individuals and universal interventions for children (given the relatively young age of prisoners) should include efforts to reduce the likelihood of unlawful behaviour.^46^ Such interventions could include social and emotional learning, and other elements that have been shown to be effective in reducing problematic behaviours and risk of self-harm.^47^

Currently, those at risk of self-harm or suicide in prisons are managed under the Assessment, Care in Custody and Teamwork (ACCT) initiative. This was piloted in 2004 and deployed across the prison estate in England and Wales during 2005 and 2007.^48^ An ACCT process can be opened for a prisoner following information from many sources that the prisoner may be at risk of self-harm or suicide. A concern form is completed by any member of staff, and this is passed to the wing or unit supervisor who begins the process. There is a meeting with the prisoner and an ACCT process is opened and an assessment done within 24 h. An ACCT case coordinator is appointed. Case interviews are done at intervals determined by the circumstances and a wide range of staff and external professionals are invited to these and a care plan is agreed.^49^

We recommend the evaluation of self-harm for all prisoners at inception to consider prior self-harm and mental disorders noted in clinical records or disclosed. Furthermore, prisoners with prior self-harm, substance use, learning difficulties and bipolar disorder, as well as those on remand, should be considered especially at risk of self-harm. Where an ACCT process is opened, case reviews should always be scheduled at shorter intervals during the first 12 months after entering prison, to reflect the elevated risk observed in this period.

Further research is needed on specific comorbidity clusters, and on a range of characteristics of self-harming behaviours, such as method, lethality, intent, frequency of and repeated self-harm; and factors including prisoners’ personality traits (e.g. impulsivity), burdens of mental health, socioeconomic status, family background and prison-specific factors which were beyond the scope of this study. We will be able to address these with a larger and deeper data-set, which will also allow us to address the comparison between remand and sentencing. All these matters will need to be tackled for policy and practice to be fully informed.

## Supporting information

DelPozo-Banos et al. supplementary material DelPozo-Banos et al. supplementary material

## Data Availability

Data are only available by application to the SAIL Databank (https://saildatabank.com/).

## Supplementary material

The supplementary material is available online at https://doi.org/10.1192/bjo.2025.10898

## Relevance statement

This study provides important insights into the prevalence and risk factors of self-harm, mental and neurodevelopmental conditions within the prison population. Our findings highlight that males in prisons are more likely to self-harm than non-prisoners even before imprisonment. While self-harm risk is highest upon imprisonment, it then declines over time to a level only slightly higher than that of non-prisoners and lower than prisoners themselves before imprisonment. We show that mental and neurodevelopmental disorders are already disproportionally present in prisoners pre-imprisonment, and are already associated with increased risk of self-harm. We further demonstrate the wide range and complexity of relationships between self-harm and mental health conditions in prisoners compared with non-prisoners, with higher rates of prior self-harm, drug use, alcohol use, depression and anxiety and mental health comorbidities in prisoners. Given these findings, self-harm prevention interventions should target people in contact with police and criminal justice systems, irrespective of imprisonment. In prisons, prevention efforts deployed at inception should target those with prior self-harm, drug use, learning difficulties, bipolar disorder and those on remand. However, universal self-harm interventions in prisons are still necessary to keep this highly vulnerable population safe.

## References

[1] Hawton K , Linsell L , Adeniji T , Sariaslan A , Fazel S. Self-harm in prisons in England and Wales: an epidemiological study of prevalence, risk factors, clustering, and subsequent suicide. Lancet 2014; 383: 1147–54. 24351319 10.1016/S0140-6736(13)62118-2 PMC3978651

[2] Wilkinson G. Sir Walter Raleigh: deliberate self-harm in the Tower of London, 1603 – Extra. Br J Psychiatry 2018; 213: 653.

[3] Favril L , Yu R , Hawton K , Fazel S. Risk factors for self-harm in prison: a systematic review and meta-analysis. Lancet Psychiatry 2020; 7: 682–91. 32711709 10.1016/S2215-0366(20)30190-5 PMC7606912

[4] Favril L , Rich JD , Hard J , Fazel S. Mental and physical health morbidity among people in prisons: an umbrella review. Lancet Public Health 2024; 9: e250–60. 38553144 10.1016/S2468-2667(24)00023-9 PMC11652378

[5] Knight B , Coid J , Ullrich S. Non-suicidal self-injury in UK prisoners. Int J Forensic Ment Health 2017; 16: 172–82.

[6] Zhong S , Senior M , Yu R , Perry A , Hawton K , Shaw J , et al. Risk factors for suicide in prisons: a systematic review and meta-analysis. Lancet Public Health 2021; 6: e164–74. 33577780 10.1016/S2468-2667(20)30233-4 PMC7907684

[7] Office for National Statistics. Drug-Related Deaths and Suicide in Offenders in the Community, England and Wales: 2011 to 2021. ONS, 2023 (https://www.ons.gov.uk/peoplepopulationandcommunity/birthsdeathsandmarriages/deaths/bulletins/drugrelateddeathsandsuicideinoffendersinthecommunityenglandandwales/2011to2021).

[8] Borschmann R , Young JT , Moran P , Spittal MJ , Snow K , Mok K , et al. Accuracy and predictive value of incarcerated adults’ accounts of their self-harm histories: findings from an Australian prospective data linkage study. CMAJ Open 2017; 5: E694–701. 10.9778/cmajo.20170058 PMC5621944 28893844

[9] Wright NMJ , Hearty P , Allgar V. Prison primary care and non-communicable diseases: a data-linkage survey of prevalence and associated risk factors. BJGP Open 2019; 3: bjgpopen19X101643. 10.3399/bjgpopen19X101643 PMC6662881 31366674

[10] Sodhi-Berry N , Preen DB , Alan J , Knuiman M , Morgan VA. Pre-sentence mental health service use by adult offenders in Western Australia: baseline results from a longitudinal whole-population cohort study. Crim Behav Ment Health 2014; 24: 204–21. 24535781 10.1002/cbm.1901

[11] Kaba F , Lewis A , Glowa-Kollisch S , Hadler J , Lee D , Alper H , et al. Solitary confinement and risk of self-harm among jail inmates. Am J Public Health 2014; 104: 442–7. 24521238 10.2105/AJPH.2013.301742 PMC3953781

[12] Jones R. Imprisonment in Wales: A Factfile. Wales Governance Centre, 2018 (http://www.walesandchestercircuit.org.uk/cmsAdmin/uploads/wgc-report-imprisonment-(finalpdf).pdf).

[13] Benchimol EI , Smeeth L , Guttmann A , Harron K , Moher D , Petersen I , et al. The REporting of studies Conducted using Observational Routinely-collected health Data (RECORD) statement. PLOS Med 2015; 12: e1001885. 26440803 10.1371/journal.pmed.1001885 PMC4595218

[14] Ford DV , Jones KH , Verplancke J-P , Lyons RA , John G , Brown G , et al. The SAIL Databank: building a national architecture for e-health research and evaluation. BMC Health Serv Res 2009; 9: 157. 19732426 10.1186/1472-6963-9-157 PMC2744675

[15] Lyons RA , Jones KH , John G , Brooks CJ , Verplancke J-P , Ford D , et al. The SAIL Databank: linking multiple health and social care datasets. BMC Med Inform Decis Mak 2009; 9: 3. 19149883 10.1186/1472-6947-9-3 PMC2648953

[16] Chisholm J. The Read clinical classification. Br Med J 1990; 300: 1092. 2344534 10.1136/bmj.300.6732.1092 PMC1662793

[17] Thayer D , Rees A , Kennedy J , Collins H , Harris D , Halcox J , et al. Measuring follow-up time in routinely-collected health datasets: challenges and solutions. PLOS One 2020; 15: e0228545. 32045428 10.1371/journal.pone.0228545 PMC7012444

[18] Marchant A , Turner S , Balbuena L , Peters E , Williams D , Lloyd K , et al. Self-harm presentation across healthcare settings by sex in young people: an e-cohort study using routinely collected linked healthcare data in Wales, UK. Arch Dis Child 2020; 105: 347–54. 31611193 10.1136/archdischild-2019-317248 PMC7146921

[19] Welsh Government. Welsh Index of Multiple Deprivation 2011 Summary Report. Welsh Government, 2011 (http://webarchive.nationalarchives.gov.uk/20120404081001/http://wales.gov.uk/topics/statistics/publications/wimd11summary/?lang=en).

[20] Cornish RP , John A , Boyd A , Tilling K , Macleod J. Defining adolescent common mental disorders using electronic primary care data: a comparison with outcomes measured using the CIS-R. BMJ Open 2016; 6: e013167. 10.1136/bmjopen-2016-013167 PMC5168670 27909036

[21] Economou A , Grey M , McGregor J , Craddock N , Lyons RA , Owen MJ , et al. The Health Informatics Cohort Enhancement project (HICE): using routinely collected primary care data to identify people with a lifetime diagnosis of psychotic disorder. BMC Res Notes 2012; 5: e95. 10.1186/1756-0500-5-95 PMC3296666 22333117

[22] John A , Friedmann Y , DelPozo-Banos M , Frizzati A , Ford T , Thapar A. Association of school absence and exclusion with recorded neurodevelopmental disorders, mental disorders, or self-harm: a nationwide, retrospective, electronic cohort study of children and young people in Wales, UK. Lancet Psychiatry 2022; 9: 23–34. 34826393 10.1016/S2215-0366(21)00367-9 PMC8674147

[23] John A , Marchant AL , Fone DL , Mcgregor JI , Dennis MS , Tan JOA , et al. Recent trends in primary-care antidepressant prescribing to children and young people: an e-cohort study. Psychol Med 2016; 46: 3315–27. 27879187 10.1017/S0033291716002099 PMC5122314

[24] John A , McGregor J , Fone D , Dunstan F , Cornish R , Lyons RA , et al. Case-finding for common mental disorders of anxiety and depression in primary care: an external validation of routinely collected data. BMC Med Inform Decis Mak 2016; 16: 35. 26979325 10.1186/s12911-016-0274-7 PMC4791907

[25] John A , McGregor J , Jones I , Lee SC , Walters JTR , Owen MJ , et al. Premature mortality among people with severe mental illness — new evidence from linked primary care data. Schizophr Res 2018; 199: 154–62. 29728293 10.1016/j.schres.2018.04.009

[26] Rees S , Watkins A , Keauffling J , John A. Incidence, mortality and survival in young people with co-occurring mental disorders and substance use: a retrospective linked routine data study in Wales. Clin Epidemiol 2022; 14: 21–38. 35058718 10.2147/CLEP.S325235 PMC8764170

[27] Thomas KH , Davies N , Metcalfe C , Windmeijer F , Martin RM , Gunnell D. Validation of suicide and self-harm records in the Clinical Practice Research Datalink. Br J Clin Pharmacol 2013; 76: 145–57. 23216533 10.1111/bcp.12059 PMC3703237

[28] Underwood JFG , Kendall KM , Berrett J , Lewis C , Anney R , van den Bree MBM , et al. Autism spectrum disorder diagnosis in adults: phenotype and genotype findings from a clinically derived cohort. Br J Psychiatry 2019; 215: 647–53. 30806336 10.1192/bjp.2019.30 PMC6949119

[29] Zou G. A modified poisson regression approach to prospective studies with binary data. Am J Epidemiol 2004; 159: 702–6. 15033648 10.1093/aje/kwh090

[30] Favril L , Shaw J , Fazel S. Prevalence and risk factors for suicide attempts in prison. Clin Psychol Rev 2022; 97: 102190. 36029609 10.1016/j.cpr.2022.102190

[31] McTernan N , Griffin E , Cully G , Kelly E , Hume S , Corcoran P. The incidence and profile of self-harm among prisoners: findings from the Self-Harm Assessment and Data Analysis Project 2017–2019. Int J Prisoner Health 2023; 19: 565–77. 10.1108/IJPH-02-2023-0012 37125411

[32] Ashworth S. Autism is underdiagnosed in prisoners. BMJ 2016; 353: i3028. 27255544 10.1136/bmj.i3028

[33] Armour C. Mental health in prison: a trauma perspective on importation and deprivation. Int J Criminol Sociol Theory 2012; 5: 886–94.

[34] Smith HP , Kaminski RJ. Inmate self-injurious behaviors: distinguishing characteristics within a retrospective study. Crim Justice Behav 2009; 37: 81–96.

[35] Hassan L , Birmingham L , Harty MA , Jarrett M , Jones P , King C , et al. Prospective cohort study of mental health during imprisonment. Br J Psychiatry 2011; 198: 37–42. 21200075 10.1192/bjp.bp.110.080333

[36] Fergusson D , Swain-Campbell N , Horwood J. How does childhood economic disadvantage lead to crime? J Child Psychol Psychiatry 2004; 45: 956–66. 15225338 10.1111/j.1469-7610.2004.t01-1-00288.x

[37] White JL , Moffitt TE , Caspi A , Bartusch DJ , Needles DJ , Stouthamer-Loeber M. Measuring impulsivity and examining its relationship to delinquency. J Abnorm Psychol 1994; 103: 192–205. 8040489 10.1037//0021-843x.103.2.192

[38] Lockwood J , Daley D , Townsend E , Sayal K. Impulsivity and self-harm in adolescence: a systematic review. Eur Child Adolesc Psychiatry 2017; 26: 387–402. 27815757 10.1007/s00787-016-0915-5 PMC5364241

[39] Singhal A , Ross J , Seminog O , Hawton K , Goldacre MJ. Risk of self-harm and suicide in people with specific psychiatric and physical disorders: comparisons between disorders using English national record linkage. J R Soc Med 2014; 107: 194–204. 24526464 10.1177/0141076814522033 PMC4023515

[40] Cybulski L , Ashcroft DM , Carr MJ , Garg S , Chew-Graham CA , Kapur N , et al. Risk factors for nonfatal self-harm and suicide among adolescents: two nested case–control studies conducted in the UK Clinical Practice Research Datalink. J Child Psychol Psychiatry 2022; 63: 1078–88. 34862981 10.1111/jcpp.13552

[41] Talbot J. Prisoners’ voices: experiences of the criminal justice system by prisoners with learning disabilities. Tizard Learn Disabil Rev 2010; 15: 33–41.

[42] Allely CS. The association of ADHD symptoms to self-harm behaviours: a systematic PRISMA review. BMC Psychiatry 2014; 14: 133. 24884622 10.1186/1471-244X-14-133 PMC4020381

[43] Young S , Cocallis KM. Attention deficit hyperactivity disorder (ADHD) in the prison system. Curr Psychiatry Rep 2019; 21: 41. 31037396 10.1007/s11920-019-1022-3

[44] Geulayov G , Casey D , McDonald KC , Foster P , Pritchard K , Wells C , et al. Incidence of suicide, hospital-presenting non-fatal self-harm, and community-occurring non-fatal self-harm in adolescents in England (the iceberg model of self-harm): a retrospective study. Lancet Psychiatry 2018; 5: 167–74. 29246453 10.1016/S2215-0366(17)30478-9

[45] Aspinall PJ. Hidden Needs: Identifying Key Vulnerable Groups in Data Collections: Vulnerable Migrants, Gypsies and Travellers, Homeless People, and Sex Workers. Centre for Health Services Studies, University of Kent, 2014 (https://assets.publishing.service.gov.uk/government/uploads/system/uploads/attachment_data/file/287805/vulnerable_groups_data_collections.pdf).

[46] Hawkins JD. Controlling crime before it happens: risk-focused prevention. Natl Inst Justice J 1995; 229: 10–8.

[47] Taylor RD , Oberle E , Durlak JA , Weissberg RP. Promoting positive youth development through school-based social and emotional learning interventions: a meta-analysis of follow-up effects. Child Dev 2017; 88: 1156–71. 28685826 10.1111/cdev.12864

[48] Humber N , Hayes A , Senior J , Fahy T , Shaw J. Identifying, monitoring and managing prisoners at risk of self-harm/suicide in England and Wales. J Forens Psychiatry Psychol 2011; 22: 22–51.

[49] Ministry of Justice and HM Prison and Probation Service. Prison Safety Policy Framework. MoJ and HM Prison and Probation Service, 2024 (https://www.gov.uk/government/publications/prison-safety-policy-framework).

