# Supplementary material for: Self-harm before and during imprisonment: cohort study of males in prison linking population-based routinely collected data in Wales
Source: BJPsych Open. 2025 Dec 12;12(1):e10. doi: 10.1192/bjo.2025.10898 (PMC12724122; doi:10.1192/bjo.2025.10898)
Supplement: DelPozo-Banos et al. supplementary material [file S2056472425108983sup001.docx]

**Supplementary file for** **Atkinson MD, DelPozo-Baños M, Lee SC, John A. Self-harm among prisoners before and during imprisonment: an e-cohort study of Welsh male prisoners linking population-based routinely collected data**

**Supplementary Methods**

*Prison system in Wales*

The current extract of MoJ data is limited to males in Welsh prisons during 2019, and to prisoners in categories B, C and D. Category A prisoners require maximum security conditions and are all housed in English prisons. Category B and C prisoners require less stringent security and category D prisoners (housed in open prisons) can be released on licence. All female prisoners residing in Wales are also housed in prisons in England and therefore excluded from our dataset.

*Tests of the proportional hazard assumption*

Tests for all the individual variables were highly significant (indicating violation of the proportional hazard assumption, Supplementary T3) except for deprivation, ASD, ADHD, learning difficulties and conduct disorder. The global test was also very highly significant.

**Supplementary Results**

*Tests of the proportional hazard assumption*

Plots of spline fits of time varying estimates of the log of the hazard ratio were shown for alcohol use, depression and anxiety in Supplementary F1. From these and similar plots for all variables, intermediate cutoff points were erected at 0.6 and 1.6 years. New analyses were carried out between these time limits.

**Supplementary T1.** The RECORD statement – checklist of items, extended from the STROBE statement, that should be reported in observational studies using routinely collected health data.

|  | **Item No.** | **STROBE items** | **Location in manuscript where items are reported** | **RECORD items** | **Location in manuscript where items are reported** |
| --- | --- | --- | --- | --- | --- |
| **Title and abstract** | | | | | |
|  | 1 | (a) Indicate the study’s design with a commonly used term in the title or the abstract (b) Provide in the abstract an informative and balanced summary of what was done and what was found |  | RECORD 1.1: The type of data used should be specified in the title or abstract. When possible, the name of the databases used should be included.  RECORD 1.2: If applicable, the geographic region and timeframe within which the study took place should be reported in the title or abstract.  RECORD 1.3: If linkage between databases was conducted for the study, this should be clearly stated in the title or abstract. | Title and Abstract |
| **Introduction** | | | | | |
| Background rationale | 2 | Explain the scientific background and rationale for the investigation being reported |  |  | Introduction |
| Objectives | 3 | State specific objectives, including any prespecified hypotheses |  |  | Introduction |
|  | | | | | |
| Study Design | 4 | Present key elements of study design early in the paper |  |  | Methods |
| Setting | 5 | Describe the setting, locations, and relevant dates, including periods of recruitment, exposure, follow-up, and data collection |  |  | Methods |
| Participants | 6 | *(a) Cohort study* - Give the eligibility criteria, and the sources and methods of selection of participants. Describe methods of follow-up  *Case-control study* - Give the eligibility criteria, and the sources and methods of case ascertainment and control selection. Give the rationale for the choice of cases and controls  *Cross-sectional study* - Give the eligibility criteria, and the sources and methods of selection of participants  *(b) Cohort study* - For matched studies, give matching criteria and number of exposed and unexposed  *Case-control study* - For matched studies, give matching criteria and the number of controls per case |  | RECORD 6.1: The methods of study population selection (such as codes or algorithms used to identify subjects) should be listed in detail. If this is not possible, an explanation should be provided.  RECORD 6.2: Any validation studies of the codes or algorithms used to select the population should be referenced. If validation was conducted for this study and not published elsewhere, detailed methods and results should be provided.  RECORD 6.3: If the study involved linkage of databases, consider use of a flow diagram or other graphical display to demonstrate the data linkage process, including the number of individuals with linked data at each stage. | Methods  Prisoner cohort  Non-prisoner cohort  Fig 1 |
| Variables | 7 | Clearly define all outcomes, exposures, predictors, potential confounders, and effect modifiers. Give diagnostic criteria, if applicable. |  | RECORD 7.1: A complete list of codes and algorithms used to classify exposures, outcomes, confounders, and effect modifiers should be provided. If these cannot be reported, an explanation should be provided. | Methods  Measures  Supplementary T2 |
| Data sources/ measurement | 8 | For each variable of interest, give sources of data and details of methods of assessment (measurement).  Describe comparability of assessment methods if there is more than one group |  |  | Methods  Measures  Supplementary T2 |
| Bias | 9 | Describe any efforts to address potential sources of bias |  |  | Methods  Data sources |
| Study size | 10 | Explain how the study size was arrived at |  |  | Methods  Prisoner cohort  Non-prisoner cohort |
| Quantitative variables | 11 | Explain how quantitative variables were handled in the analyses. If applicable, describe which groupings were chosen, and why |  |  | Methods  Measures |
| Statistical methods | 12 | (a) Describe all statistical methods, including those used to control for confounding  (b) Describe any methods used to examine subgroups and interactions  (c) Explain how missing data were addressed  (d) *Cohort study* - If applicable, explain how loss to follow-up was addressed  *Case-control study* - If applicable, explain how matching of cases and controls was addressed  *Cross-sectional study* - If applicable, describe analytical methods taking account of sampling strategy  (e) Describe any sensitivity analyses |  |  | Methods  Analyses  Supplementary T3  Supplementary F1 |
| Data access and cleaning methods |  | .. |  | RECORD 12.1: Authors should describe the extent to which the investigators had access to the database population used to create the study population.  RECORD 12.2: Authors should provide information on the data cleaning methods used in the study. | Methods  Data sources  Methods  Data sources |
| Linkage |  | .. |  | RECORD 12.3: State whether the study included person-level, institutional-level, or other data linkage across two or more databases. The methods of linkage and methods of linkage quality evaluation should be provided. | Methods  Data sources |
| **Results** | | | | | |
| Participants | 13 | (a) Report the numbers of individuals at each stage of the study (*e.g.*, numbers potentially eligible, examined for eligibility, confirmed eligible, included in the study, completing follow-up, and analysed)  (b) Give reasons for non-participation at each stage.  (c) Consider use of a flow diagram |  | RECORD 13.1: Describe in detail the selection of the persons included in the study (*i.e.,* study population selection) including filtering based on data quality, data availability and linkage. The selection of included persons can be described in the text and/or by means of the study flow diagram. | Results  Supplementary F2 |
| Descriptive data | 14 | (a) Give characteristics of study participants (*e.g.*, demographic, clinical, social) and information on exposures and potential confounders  (b) Indicate the number of participants with missing data for each variable of interest  (c) *Cohort study* - summarise follow-up time (*e.g.*, average and total amount) |  |  | Results  Demographics of prisoners  Supplementary T4 |
| Outcome data | 15 | *Cohort study* - Report numbers of outcome events or summary measures over time  *Case-control study* - Report numbers in each exposure category, or summary measures of exposure  *Cross-sectional study* - Report numbers of outcome events or summary measures |  |  | Results  Comparison between prisoners and the non-prisoner population |
| Main results | 16 | (a) Give unadjusted estimates and, if applicable, confounder-adjusted estimates and their precision (e.g., 95% confidence interval). Make clear which confounders were adjusted for and why they were included  (b) Report category boundaries when continuous variables were categorized  (c) If relevant, consider translating estimates of relative risk into absolute risk for a meaningful time period |  |  | Results  Table 1-2, Supplementary T5-T6  Fig 2 and Supplementary F3-F4 |
| Other analyses | 17 | Report other analyses done—e.g., analyses of subgroups and interactions, and sensitivity analyses |  |  |  |
| **Discussion** | | | | | |
| Key results | 18 | Summarise key results with reference to study objectives |  |  | Discussion |
| Limitations | 19 | Discuss limitations of the study, taking into account sources of potential bias or imprecision. Discuss both direction and magnitude of any potential bias |  | RECORD 19.1: Discuss the implications of using data that were not created or collected to answer the specific research question(s). Include discussion of misclassification bias, unmeasured confounding, missing data, and changing eligibility over time, as they pertain to the study being reported. | Discussion  Strengths and limitations |
| Interpretation | 20 | Give a cautious overall interpretation of results considering objectives, limitations, multiplicity of analyses, results from similar studies, and other relevant evidence |  |  | Discussion |
| Generalisability | 21 | Discuss the generalisability (external validity) of the study results |  |  | Discussion |
| **Other InformationFunding** | | | | | |
| Funding | 22 | Give the source of funding and the role of the funders for the present study and, if applicable, for the original study on which the present article is based |  |  | Funding |
| Accessibility of protocol, raw data, and programming code |  | .. |  | RECORD 22.1: Authors should provide information on how to access any supplemental information such as the study protocol, raw data, or programming code. | Data availability |

*Reference: Benchimol EI, Smeeth L, Guttmann A, Harron K, Moher D, Petersen I, Sørensen HT, von Elm E, Langan SM, the RECORD Working Committee. The REporting of studies Conducted using Observational Routinely-collected health Data (RECORD) Statement. *PLoS Medicine* 2015; in press.

*Checklist is protected under Creative Commons Attribution ([CC BY](http://creativecommons.org/licenses/by/4.0/)) license.

**Supplementary T2.** List of measurements used and references to code lists and algorithms used to ascertain them from primary care, emergency presentations and hospital admissions.

| Variable | Primary care | Emergency department | Hospital admissions |
| --- | --- | --- | --- |
| Self-harm | Algorithm [1,2] | Algorithm [1] | Algorithm [1] |
| Alcohol abuse | C2944 [3] <https://conceptlibrary.saildatabank.com/concepts/C2944/version/8650/detail/> | Diagnoses and alcohol flag, other codes^a^ | C2946 [3] <https://conceptlibrary.saildatabank.com/concepts/C2946/version/8643/detail/> |
| Drug use | C2945 [3] <https://conceptlibrary.saildatabank.com/concepts/C2945/version/8649/detail/> | Diagnoses and other codes^b^ | C2947 [3] <https://conceptlibrary.saildatabank.com/concepts/C2947/version/8644/detail/> |
| Depression | Algorithm [4,5,6] |  | Diagnoses C2942 [5] <https://conceptlibrary.saildatabank.com/concepts/C2942/version/10167/detail/> |
| Anxiety | Algorithm [4,5,6] |  | Diagnoses C2941 [4] <https://conceptlibrary.saildatabank.com/concepts/C2941/version/8645/detail/> |
| Attention Deficit hyperactivity disorder | C2708 [7] <https://conceptlibrary.saildatabank.com/concepts/C2708/version/9951/detail/> |  | C2931 [7] <https://conceptlibrary.saildatabank.com/concepts/C2931/version/9919/detail/> |
| ASD | C2709 [8]  <https://conceptlibrary.saildatabank.com/concepts/C2709/version/9884/detail/> |  | C2930 [8]  <https://conceptlibrary.saildatabank.com/concepts/C2930/version/9883/detail/> |
| Learning difficulties | C2711 [7] <https://conceptlibrary.saildatabank.com/concepts/C2711/version/8657/detail/> |  | C2940 [7] <https://conceptlibrary.saildatabank.com/concepts/C2940/version/8658/detail/> |
| Conduct disorder | C2710 [7] <https://conceptlibrary.saildatabank.com/concepts/C2710/version/9894/detail/> |  | C2933 [7] <https://conceptlibrary.saildatabank.com/concepts/C2933/version/9895/detail/> |
| Bipolar disorder and other mood disorders | C2714 [9] <https://conceptlibrary.saildatabank.com/concepts/C2714/version/8444/detail/> |  | C2932 [9] <https://conceptlibrary.saildatabank.com/concepts/C2932/version/8648/detail/> |
| Schizophrenia | C2716 [9] <https://conceptlibrary.saildatabank.com/concepts/C2716/version/8446/detail/> |  | C2939 [9] <https://conceptlibrary.saildatabank.com/concepts/C2939/version/8647/detail/> |
| Other psychotic disorders and severe mental illness | C3160 [9] <https://conceptlibrary.saildatabank.com/concepts/C3160/version/8652/detail/> |  | C3159 [10] <https://conceptlibrary.saildatabank.com/concepts/C3159/version/8651/detail/> |

^a^ For alcohol abuse we include two EDDS codes for alcohol poisoning or overdose and for chronic alcohol abuse. We also use 7 ICD 10 codes with 3 characters and an alcohol indicator code.

^b^ For drug use we include 4 EDDS diagnosis codes for poisoning or overdose, 8 ICD 10 codes of length 3, and a chronic drug abuse flag.

*References:*

[1] Marchant A, Turner S, Balbuena L, Peters E, Williams D, Lloyd K, et al. Self-harm presentation across healthcare settings by sex in young people: an e-cohort study using routinely collected linked healthcare data in Wales, UK. *Arch Dis Child* 2020; **105**: 347-54.

[2] Thomas KH, Davies N, Metcalfe C, Windmeijer F, Martin RM, Gunnell D. Validation of suicide and self-harm records in the Clinical Practice Research Datalink. *Br J Clin Pharmacol* 2013; **76**:145-57.

[3] Rees S, Watkins A, Keauffling J, John A. Incidence, Mortality and Survival in Young People with Co-Occurring Mental Disorders and Substance Use: A Retrospective Linked Routine Data Study in Wales. *Clin Epidemiol* 2022; **14**: 21-38.

[4] Cornish RP, John A, Boyd A, Tilling K, Macleod J. Defining adolescent common mental disorders using electronic primary care data: a comparison with outcomes measured using the CIS-R. *BMJ Open* 2016; **6**; e013167.

[5. John A, Marchant AL, Fone DL, McGregor JI, Dennis MS, Tan JOA, et al. Recent trends in primary-care antidepressant prescribing to children and young people: an e-cohort study. *Psychol Med* 2016; **46**: 3315-27.

[6. John A, McGregor J, Fone D, Dunstan F, Cornish R, Lyons RA, et al. Case-finding for common mental disorders of anxiety and depression in primary care: an external validation of routinely collected data. *BMC Med Inform Decis Mak* 2016; **16**: 35.

[7] John A, Friedmann Y, DelPozo-Banos M, Frizzati A, Ford T, Thapar A: Association of school absence and exclusion with recorded neurodevelopmental disorders, mental disorders, or self-harm: a nationwide, retrospective, electronic cohort study of children and young people in Wales, UK. *Lancet Psychiatry* 2022; **9**: 23-34.

[8] Underwood JFG, Kendall KM, Berrett J, Lewis C, Anney R, van den Bree MBM, et al. Autism spectrum disorder diagnosis in adults: phenotype and genotype findings from a clinically derived cohort. *Br J Psychiatry* 2019; **215**: 647-53

[9] John A, McGregor J, Jones I, Lee SC, Walters JTR, Owen MJ et al. Premature mortality among people with severe mental illness-new evidence from linked primary care data. *Schizophrenia Res* 2018; **199**: 154-162.

[10] Economou A, Grey M, McGregor J, Craddock N, Lyons RA, Owen MJ et al. The Health Informatics Cohort Enhancement project (HICE): using routinely collected primary care data to identify people with a lifetime diagnosis of psychotic disorder. *BMC Res Notes* 2012; **5**: 95.

**Supplementary T3.** Output from the test for violation of the proportional hazard assumption for the full dataset.

| Variable | Chi-square | df | p-value |
| --- | --- | --- | --- |
| Group | 449.0 | 3 | <0.0001 |
| Age class | 32.5 | 2 | <0.0001 |
| Deprivation | 4.2 | 4 | 0.376 |
| Self-harm | 167.0 | 1 | <0.0001 |
| Alcohol use | 68.2 | 1 | <0.0001 |
| Drug use | 73.0 | 1 | <0.0001 |
| Depression | 161.0 | 1 | <0.0001 |
| Anxiety | 139.0 | 1 | <0.0001 |
| ADHD | 4.7 | 1 | 0.03 |
| ASD | 6.4 | 1 | 0.011 |
| Learning difficulties | 1.5 | 1 | 0.223 |
| Conduct disorder | 0.0 | 1 | 0.981 |
| Bipolar disorder | 16.5 | 1 | <0.0001 |
| Schizophrenia | 22.3 | 1 | <0.0001 |
| Other psychotic disorders | 35.5 | 1 | <0.0001 |
| Group × Age class | 297.0 | 6 | <0.0001 |
| Group × Deprivation | 415.0 | 12 | <0.0001 |
| Group × Self-harm | 326.0 | 3 | <0.0001 |
| Group × Alcohol use | 211.0 | 3 | <0.0001 |
| Group × Drug use | 250.0 | 3 | <0.0001 |
| Group × Depression | 394.0 | 3 | <0.0001 |
| Group × Anxiety | 320.0 | 3 | <0.0001 |
| Group × ADHD | 57.8 | 3 | <0.0001 |
| Group × ASD | 27.5 | 3 | <0.0001 |
| Group × Learning difficulties | 11.0 | 3 | 0.012 |
| Group × Conduct disorder | 28.0 | 3 | <0.0001 |
| Group × Bipolar disorder | 36.4 | 3 | <0.0001 |
| Group × Schizophrenia | 46.4 | 3 | <0.0001 |
| Group × Other psychotic disorders | 48.0 | 3 | <0.0001 |
| Global test | 733.0 | 75 | <0.0001 |

**Supplementary T4.** Demographics and health history of prisoners and non-prisoners prior to their imprisonment/index date.

|  | Prisoners | Non-prisoners |  |  | |
| --- | --- | --- | --- | --- | --- |
| Variable | N (%) | N (%) | Diff (95% CI) | Chi-square | |
| Total | 4081 | 450748 |  | - | |
| Age | | | | | |
| <25 | 924 (22.6) | 104063 (23.1) | -0.4 (-1.7, 0.9) | 0.4 | NS |
| 25-64 | 3064 (75.1) | 333167 (73.9) | 1.2 (-0.2, 2.5) | 2.8 | NS |
| 65+ | 102 (2.5) | 13518 (3.0) | -0.7 (-1.2, -0.2) | 7.0 | ** |
| WIMD Quintile | | | | | |
| Quintile 1 | 245 (6.0) | 84175 (18.7) | -12.7 (-13.4, -11.9) | 428.7 | *** |
| Quintile 2 | 328 (8.0) | 78724 (17.5) | -9.4 (-10.3, -8.6) | 249.7 | *** |
| Quintile 3 | 552 (13.5) | 91891 (20.4) | -6.9 (-7.9, -5.8) | 117.1 | ** |
| Quintile 4 | 947 (23.2) | 95202 (21.1) | 2.1 (0.8, 3.4) | 10.4 | *** |
| Quintile 5 (most deprived) | 2009 (49.2) | 100756 (22.4) | 26.9 (25.3, 28.4) | 1668.7 | *** |
| Health history before the imprisonment/index date | | | | | |
| Self-harm | 1623 (39.8) | 25459 (5.6) | 34.1 (32.6, 35.6) | 8402.8 | *** |
| Alcohol use | 1531 (37.5) | 36254 (8) | 29.5 (28.0, 31.0) | 4608.0 | *** |
| Drug use | 2146 (52.6) | 26124 (5.8) | 46.8 (45.2, 48.3) | 15181.3 | *** |
| Depression | 1989 (48.7) | 98327 (21.8) | 38.1 (36.6, 39.6) | 3397.0 | *** |
| Anxiety | 1857 (45.5) | 78780 (17.5) | 28.0 (26.5, 29.6) | 2176.0 | *** |
| ADHD | 377 (9.2) | 7525 (1.7) | 7.6 (6.7, 8.5) | 1352.6 | *** |
| Autism spectrum disorder | 38 (0.9) | 4746 (1.1) | -0.1 (-0.4, 0.2) | 0.5 | NS |
| Learning difficulties | 73 (1.8) | 5912 (1.3) | 0.5 (0.1, 0.9) | 6.7 | ** |
| Conduct disorder | 294 (7.2) | 6222 (1.4) | 5.8 (5.0, 6.6) | 967.3 | *** |
| Bipolar disorder | 44 (1.1) | 1538 (0.3) | 0.7 (0.4, 1.1) | 61.3 | *** |
| Schizophrenia | 305 (7.5) | 4253 (0.9) | 6.5 (5.7, 7.3) | 1731.8 | *** |
| Other psychotic disorders | 172 (4.2) | 3690 (0.8) | 3.4 (2.8, 4.0) | 550.0 | *** |
| Number of mental health comorbidities | | | | | |
| 0 | 699 (17.1) | 289658(64.3) | -47.1 (-48.3, -46) | 3890.1 | *** |
| 1 | 638(15.6) | 81061(18) | -2.4 (-3.5, -1.2) | 15.0 | *** |
| 2 to 5 | 2443(59.9) | 77939(17.3) | 42.6 (41.1, 44.1) | 5034.9 | *** |
| More than 5 | 301(7.4) | 2090(0.5) | 6.9 (6.1, 7.7) | 3681.8 | *** |
| Health history after the imprisonment/index date^a^ | | | | | |
| Self-harm | 402 (10.6) | 3196 (0.7) | 9.9 (8.9, 10.9) | 1142.4 | *** |

^a^ Based on the subset of 3,780 prisoners and 428,017 non-prisoners used in the Cox regression analysis.

Diff: Difference in % points (Prisoners - Non-prisoners). Significance codes for chi-squared test are: P-value ≤0.0001 ***, P-value ≤0.001 **, P-value ≤ 0.01 *, P-value ≤ 0.05 ‡, P-value >0.05 NS.

**Supplementary T5.** Summary (OR and 95% CIs) of the nested logistic regression analysis of health history variable ascertained before imprisonment/index date. The dependent variable is non-prisoners (no) or prisoner (yes).

|  | Model 1 | | Model 2 | | Model 3 | | Model 4 | | Model 5 | | Model 6 | |
| --- | --- | --- | --- | --- | --- | --- | --- | --- | --- | --- | --- | --- |
| WIMD quintile (reference: Quintile 1) | | | | | | | | | | | | |
| Quintile 2 | 1.4 (1.2, 1.7) | *** | 1.3 (1.1, 1.6) | *** | 1.3 (1.1, 1.5) | ** | 1.3 (1.1, 1.5) | ** | 1.3 (1.1, 1.5) | ** | 1.3 (1.1, 1.5) | ** |
| Quintile 3 | 2.1 (1.8, 2.4) | *** | 1.8 (1.6, 2.1) | *** | 1.7 (1.4, 1.2) | *** | 1.6 (1.4, 1.9) | *** | 1.6 (1.4, 1.9) | *** | 1.6 (1.4, 1.9) | *** |
| Quintile 4 | 3.4 (3.0, 3.9) | *** | 2.8 (2.4, 3.2) | *** | 2.3 (2.0, 2.7) | *** | 2.3 (2.0, 2.6) | *** | 2.2 (1.9, 2.6) | *** | 2.2 (1.9, 2.6) | *** |
| Quintile 5 | 6.9 (6.0, 7.8) | *** | 5.0 (4.4, 5.7) | *** | 3.9 (3.4, 4.4) | *** | 3.7 (3.2, 4.2) | *** | 3.7 (3.2, 4.2) | *** | 3.6 (3.2, 4.2) | *** |
| Self-harm | - | | 9.0 (8.4, 9.6) | *** | 2.5 (2.3, 2.7) | *** | 2.2 (2.0, 2.4) | *** | 2.1 (1.9, 2.2) | *** | 2.1 (1.9, 2.2) | *** |
| Alcohol use | - | | - | | 1.6 (1.5, 1.7) | *** | 1.5 (1.4, 1.6) | *** | 1.5 (1.4, 1.6) | *** | 1.5 (1.4, 1.6) | *** |
| Drug use | - | | - | | 8.0 (7.4, 8.6) | *** | 6.6 (6.1, 7.1) | *** | 6.2 (5.7, 6.8) | *** | 6.2 (5.7, 6.7) | *** |
| Depression | - | | - | | - | | 1.7 (1.6, 1.8) | *** | 1.7 (1.6, 1.9) | *** | 1.7 (1.6, 1.9) | ** |
| Anxiety | - | | - | | - | | 1.2 (1.1, 1.3) | *** | 1.2 (1.1, 1.3) | *** | 1.2 (1.1, 1.3) | *** |
| ADHD | - | | - | | - | | - | | 2.4 (2.2, 2.8) | *** | 2.4 (2.2, 2.8) | *** |
| ASD | - | | - | | - | | - | | 0.5 (0.4, 0.7) | *** | 0.5 (0.4, 0.7) | *** |
| Learning difficulties | - | | - | | - | | - | | 0.7 (0.6, 0.9) | ** | 0.7 (0.6, 0.9) | ** |
| Conduct disorder | - | | - | | - | | - | | 1.6 (1.4, 1.8) | *** | 1.6 (1.4, 1.8) | *** |
| Bipolar disorder | - | | - | | - | | - | | - | | 0.5 (0.4, 0.7) | *** |
| Schizophrenia | - | | - | | - | | - | | - | | 1.4 (1.2, 1.6) | *** |
| Other psychotic disorders | - | | - | | - | | - | | - | | 0.8 (0.7, 1.0) | * |

Significance codes are: P-value ≤0.0001 ***, P-value ≤0.001 **, P-value ≤0.01 *, P-value ≤ 0.05 ‡, P-value >0.05 NS. OR>1 indicates a variable is more common in prisoners than non-prisoners.

**Supplementary T6.** Summary (counts, RR and 95% CIs) of subsequent self-harm in prisoners and non-prisoners after the imprisonment/index date and in the 3 previous years (‘-3 years’).

| Date | Variable | Prisoners |  | Non-prisoners |  | Prisoners/Non-prisoners | |
| --- | --- | --- | --- | --- | --- | --- | --- |
|  |  | N (%) |  | N (%) |  | RR (95% CI)^b^ | Sig.^a^ |
| Imprisonment/index date | Total | 3,780 |  | 428,017 |  |  |  |
|  | Prior self-harm | 1,499 (39.7) |  | 24,496 |  |  |  |
|  | Subsequent self-harm | 402 (10.6) |  | 3,196 (0.7) |  | 5.80 (5.25, 6.41) | ‡ |
| Imprisonment/index date – 3 years | Total | 3,366 |  | 381,340 |  |  |  |
|  | Prior self-harm | 1,009 (30) |  | 18,326 (4.8) |  |  |  |
|  | Subsequent self-harm | 681 (20.2) |  | 5,412 (1.4) |  | 6.02 (5.60, 6.47) | ‡ |
|  | Difference^c^ |  |  |  |  | 0.96 (0.85, 1.09) | NS |

^a^ Significance: P-value ≤0.05 ‡, P-value >0.05 NS.

^b^ Risk Ratio (RR) adjusted for age group, deprivation, and the interaction between prisoner/non-prisoner and prior self-harm. RR is >1 if subsequent self-harm risk in ‘prisoners’ is greater than in ‘non-prisoners’, =1 if equal, and <1 if lower.

^c^ The interaction term between cohort and time period measuring how RR in the prison population changed when in prison compared to before incarceration in relation to the non-prison population population.

N (%): Number of people (‘prisoners’ or ‘non-prisoners’) and proportion.

**Supplementary T7.** Summary (HR and 95% CIs) of associations between previous self-harm/mental health conditions and subsequent self-harm in ‘prisoners’, ‘non-prisoners’, ‘prisoners - 3 years’ and ‘non-prisoners - 3 years’ groups in three follow-up periods.

|  |  |  | Period 1 (0-0.6 year) | | |  | Period 2 (0.6-1.6 year) | | |  | Period 3 (1.6-3 year) | | |
| --- | --- | --- | --- | --- | --- | --- | --- | --- | --- | --- | --- | --- | --- |
| Group | Variable | Category | HR | 95% CI | Sig.^a^ |  | HR | 95% CI | Sig.^a^ |  | HR | 95% CI | Sig.^a^ |
| prisoners | Age (ref.: <25) | Age 25-64 | 0.4 | (0.3 - 0.5) | ‡ |  | 0.4 | (0.3 - 0.7) | ‡ |  | 0.3 | (0.2 - 0.5) | ‡ |
|  |  | Age 65+ | 0.4 | (0.1 - 1.4) | NS |  | -^b^ | - | - |  | - | - | - |
|  | WIMD quintile (ref.: Quintile 1) (Quintile 5:  most deprived) | Quintile 2 | 1.0 | (0.5 - 2.3) | NS |  | 1.1 | (0.3 - 4.7) | NS |  | 2.4 | (0.5 - 11.8) | NS |
|  |  | Quintile 3 | 1.1 | (0.5 - 2.3) | NS |  | 1.5 | (0.4 - 5.4) | NS |  | 0.9 | (0.2 - 5.2) | NS |
|  |  | Quintile 4 | 1.3 | (0.7 - 2.5) | NS |  | 1.5 | (0.4 - 5.0) | NS |  | 1.5 | (0.3 - 6.6) | NS |
|  |  | Quintile 5 | 1.4 | (0.8 - 2.7) | NS |  | 1.8 | (0.6 - 5.9) | NS |  | 2.3 | (0.6 - 9.6) | NS |
|  | Self-harm | Yes | 2.3 | (1.7 - 3.1) | ‡ |  | 1.7 | (1.0 - 2.7) | ‡ |  | 1.2 | (0.7 - 2.1) | NS |
|  | Alcohol use | Yes | 1.1 | (0.8 - 1.5) | NS |  | 1.2 | (0.8 - 2.0) | NS |  | 1.6 | (0.9 - 2.8) | NS |
|  | Drug use | Yes | 1.5 | (1.1 - 2.1) | ‡ |  | 3.0 | (1.7 - 5.5) | ‡ |  | 2.2 | (1.2 - 4.0) | ‡ |
|  | Depression | Yes | 1.0 | (0.7 - 1.4) | NS |  | 1.2 | (0.7 - 2.0) | NS |  | 1.1 | (0.6 - 2.0) | NS |
|  | Anxiety | Yes | 1.3 | (1.0 - 1.7) | NS |  | 1.0 | (0.6 - 1.6) | NS |  | 1.3 | (0.8 - 2.3) | NS |
|  | ADHD | Yes | 1.7 | (1.2 - 2.4) | ‡ |  | 2.1 | (1.2 - 3.7) | ‡ |  | 1.1 | (0.5 - 2.2) | NS |
|  | ASD | Yes | 2.4 | (1.2 - 4.7) | ‡ |  | - | - | - |  | - | - | - |
|  | Learning difficulties | Yes | 1.9 | (1.0 - 3.5) | ‡ |  | 3.1 | (1.1 - 8.6) | ‡ |  | 1.9 | (0.3 - 14.4) | NS |
|  | Conduct disorder | Yes | 1.4 | (0.9 - 2.0) | NS |  | 0.9 | (0.4 - 2.0) | NS |  | 2.7 | (1.3 - 5.2) | ‡ |
|  | Bipolar disorder | Yes | 1.9 | (0.9 - 3.8) | NS |  | 2.5 | (0.6 - 10.8) | NS |  | 2.4 | (0.2 - 26.7) | NS |
|  | Schizophrenia | Yes | 1.1 | (0.7 - 1.7) | NS |  | 1.3 | (0.6 - 2.7) | NS |  | 2.0 | (0.8 - 5.1) | NS |
|  | Other psychotic conditions | Yes | 1.1 | (0.6 - 2.0) | NS |  | 0.6 | (0.2 - 1.9) | NS |  | 0.3 | (0.1 - 2.1) | NS |
|  |  |  |  |  |  |  |  |  |  |  |  |  |  |
| prisoners - 3 years | Age (ref.: <25) | Age 25-64 | 1.3 | (0.8 - 2.1) | NS |  | 1.6 | (0.9 - 2.9) | NS |  | 2.5 | (1.4 - 4.7) | ‡ |
|  |  | Age 65+ | 1.0 | (0.1 - 9.6) | NS |  | - | - | - |  | - | - | - |
|  | WIMD quintile (ref.: Quintile 1) (Quintile 5:  most deprived) | Quintile 2 | 1.2 | (0.3 - 4.3) | NS |  | 1.0 | (0.2 - 5.3) | NS |  | 0.6 | (0.1 - 3.4) | NS |
|  |  | Quintile 3 | 1.0 | (0.3 - 3.2) | NS |  | 0.8 | (0.2 - 3.6) | NS |  | 1.3 | (0.2 - 7.7) | NS |
|  |  | Quintile 4 | 0.9 | (0.3 - 2.6) | NS |  | 0.9 | (0.2 - 3.4) | NS |  | 0.8 | (0.2 - 3.8) | NS |
|  |  | Quintile 5 | 0.8 | (0.3 - 2.3) | NS |  | 0.5 | (0.1 - 2.0) | NS |  | 0.6 | (0.1 - 2.5) | NS |
|  | Self-harm | Yes | 2.6 | (1.5 - 4.5) | ‡ |  | 1.8 | (1.0 - 3.3) | ‡ |  | 1.5 | (0.8 - 2.7) | NS |
|  | Alcohol use | Yes | 1.2 | (0.8 - 2.0) | NS |  | 1.0 | (0.6 - 1.8) | NS |  | 0.8 | (0.4 - 1.5) | NS |
|  | Drug use | Yes | 1.0 | (0.6 - 1.8) | NS |  | 0.4 | (0.2 - 0.9) | ‡ |  | 0.6 | (0.3 - 1.2) | NS |
|  | Depression | Yes | 1.3 | (0.7 - 2.1) | NS |  | 1.0 | (0.5 - 1.8) | NS |  | 1.1 | (0.6 - 2.2) | NS |
|  | Anxiety | Yes | 0.9 | (0.5 - 1.4) | NS |  | 0.9 | (0.5 - 1.6) | NS |  | 0.8 | (0.4 - 1.4) | NS |
|  | ADHD | Yes | 0.8 | (0.4 - 1.4) | NS |  | 0.6 | (0.3 - 1.2) | NS |  | 1.2 | (0.5 - 2.7) | NS |
|  | ASD | Yes | 0.8 | (0.2 - 3.7) | NS |  | - | - | - |  | - | - | - |
|  | Learning difficulties | Yes | 0.2 | (0.0 - 1.4) | NS |  | 0.5 | (0.1 - 1.7) | NS |  | 0.4 | (0.0 - 3.6) | NS |
|  | Conduct disorder | Yes | 0.4 | (0.2 - 0.8) | ‡ |  | 1.3 | (0.5 - 3.2) | NS |  | 0.4 | (0.2 - 0.8) | ‡ |
|  | Bipolar disorder | Yes | 1.3 | (0.4 - 4.2) | NS |  | 0.7 | (0.1 - 4.5) | NS |  | 1.0 | (0.1 - 13.5) | NS |
|  | Schizophrenia | Yes | 1.3 | (0.6 - 2.6) | NS |  | 0.9 | (0.4 - 2.3) | NS |  | 0.6 | (0.2 - 1.8) | NS |
|  | Other psychotic conditions | Yes | 1.2 | (0.5 - 2.9) | NS |  | 1.7 | (0.4 - 7.2) | NS |  | 1.7 | (0.2 - 11.9) | NS |
|  |  |  |  |  |  |  |  |  |  |  |  |  |  |
| non-prisoners | Age (ref.: <25) | Age 25-64 | 0.9 | (0.7 - 1.3) | NS |  | 0.7 | (0.4 - 1.2) | NS |  | 1.5 | (0.9 - 2.7) | NS |
|  |  | Age 65+ | 0.2 | (0.0 - 0.8) | ‡ |  | - | - | - |  | - | - | - |
|  | WIMD quintile (ref.: Quintile 1) (Quintile 5:  most deprived) | Quintile 2 | 1.3 | (0.6 - 3.0) | NS |  | 1.1 | (0.3 - 4.9) | NS |  | 0.5 | (0.1 - 2.3) | NS |
|  |  | Quintile 3 | 1.4 | (0.7 - 3.0) | NS |  | 0.9 | (0.2 - 3.2) | NS |  | 1.6 | (0.3 - 9.1) | NS |
|  |  | Quintile 4 | 1.3 | (0.6 - 2.6) | NS |  | 1.2 | (0.4 - 4.2) | NS |  | 1.2 | (0.3 - 5.6) | NS |
|  |  | Quintile 5 | 1.3 | (0.7 - 2.6) | NS |  | 0.9 | (0.3 - 3.1) | NS |  | 0.8 | (0.2 - 3.4) | NS |
|  | Self-harm | Yes | 1.9 | (1.3 - 2.7) | ‡ |  | 1.9 | (1.2 - 3.2) | ‡ |  | 2.3 | (1.3 - 4.2) | ‡ |
|  | Alcohol use | Yes | 1.8 | (1.3 - 2.5) | ‡ |  | 1.8 | (1.1 - 2.9) | ‡ |  | 1.2 | (0.7 - 2.2) | NS |
|  | Drug use | Yes | 1.4 | (1.0 - 2.0) | NS |  | 0.7 | (0.4 - 1.3) | NS |  | 0.9 | (0.5 - 1.7) | NS |
|  | Depression | Yes | 2.8 | (2.0 - 4.0) | ‡ |  | 2.3 | (1.4 - 4.0) | ‡ |  | 2.1 | (1.2 - 3.9) | ‡ |
|  | Anxiety | Yes | 1.0 | (0.7 - 1.4) | NS |  | 1.2 | (0.8 - 2.0) | NS |  | 0.7 | (0.4 - 1.2) | NS |
|  | ADHD | Yes | 1.2 | (0.8 - 1.8) | NS |  | 0.7 | (0.4 - 1.3) | NS |  | 1.8 | (0.8 - 4.0) | NS |
|  | ASD | Yes | 0.7 | (0.3 - 1.3) | NS |  | - | - | - |  | - | - | - |
|  | Learning difficulties | Yes | 0.6 | (0.3 - 1.2) | NS |  | 0.5 | (0.2 - 1.5) | NS |  | 0.8 | (0.1 - 5.9) | NS |
|  | Conduct disorder | Yes | 0.8 | (0.5 - 1.2) | NS |  | 1.6 | (0.7 - 3.8) | NS |  | 0.5 | (0.2 - 1.0) | ‡ |
|  | Bipolar disorder | Yes | 0.9 | (0.4 - 2.0) | NS |  | 0.4 | (0.1 - 1.7) | NS |  | 0.5 | (0.0 - 5.6) | NS |
|  | Schizophrenia | Yes | 1.3 | (0.8 - 2.2) | NS |  | 1.0 | (0.4 - 2.2) | NS |  | 0.9 | (0.3 - 2.5) | NS |
|  | Other psychotic conditions | Yes | 1.0 | (0.6 - 1.9) | NS |  | 2.2 | (0.6 - 7.9) | NS |  | 2.4 | (0.4 - 15.4) | NS |
|  |  |  |  |  |  |  |  |  |  |  |  |  |  |
| non-prisoners - 3 years | Age (ref.: <25) | Age 25-64 | 0.8 | (0.6 - 1.1) | NS |  | 0.8 | (0.5 - 1.3) | NS |  | 1.3 | (0.7 - 2.3) | NS |
|  |  | Age 65+ | 0.1 | (0.0 - 0.6) | ‡ |  | - | - | - |  | - | - | - |
|  | WIMD quintile (ref.: Quintile 1) (Quintile 5:  most deprived) | Quintile 2 | 1.2 | (0.5 - 2.8) | NS |  | 1.2 | (0.3 - 5.1) | NS |  | 0.5 | (0.1 - 2.3) | NS |
|  |  | Quintile 3 | 1.1 | (0.5 - 2.4) | NS |  | 0.9 | (0.3 - 3.5) | NS |  | 1.6 | (0.3 - 9.2) | NS |
|  |  | Quintile 4 | 1.3 | (0.6 - 2.7) | NS |  | 1.1 | (0.3 - 3.7) | NS |  | 1.2 | (0.3 - 5.6) | NS |
|  |  | Quintile 5 | 1.1 | (0.6 - 2.3) | NS |  | 1.0 | (0.3 - 3.3) | NS |  | 0.9 | (0.2 - 3.7) | NS |
|  | Self-harm | Yes | 2.1 | (1.5 - 3.0) | ‡ |  | 2.0 | (1.2 - 3.4) | ‡ |  | 2.7 | (1.5 - 4.8) | ‡ |
|  | Alcohol use | Yes | 1.7 | (1.2 - 2.4) | ‡ |  | 1.5 | (0.9 - 2.4) | NS |  | 1.1 | (0.6 - 2.0) | NS |
|  | Drug use | Yes | 1.4 | (1.0 - 2.0) | NS |  | 0.8 | (0.4 - 1.4) | NS |  | 0.9 | (0.5 - 1.8) | NS |
|  | Depression | Yes | 2.7 | (1.9 - 3.8) | ‡ |  | 2.1 | (1.2 - 3.5) | ‡ |  | 2.0 | (1.1 - 3.6) | ‡ |
|  | Anxiety | Yes | 1.1 | (0.8 - 1.6) | NS |  | 1.5 | (0.9 - 2.4) | NS |  | 0.9 | (0.5 - 1.5) | NS |
|  | ADHD | Yes | 0.9 | (0.6 - 1.4) | NS |  | 0.9 | (0.5 - 1.6) | NS |  | 1.7 | (0.8 - 3.6) | NS |
|  | ASD | Yes | 0.6 | (0.3 - 1.2) | NS |  | - | - | - |  | - | - | - |
|  | Learning difficulties | Yes | 0.8 | (0.4 - 1.6) | NS |  | 0.5 | (0.2 - 1.3) | NS |  | 0.6 | (0.1 - 4.8) | NS |
|  | Conduct disorder | Yes | 1.0 | (0.6 - 1.5) | NS |  | 1.4 | (0.6 - 3.3) | NS |  | 0.6 | (0.3 - 1.2) | NS |
|  | Bipolar disorder | Yes | 0.7 | (0.3 - 1.5) | NS |  | 0.4 | (0.1 - 1.9) | NS |  | 0.6 | (0.1 - 6.4) | NS |
|  | Schizophrenia | Yes | 1.4 | (0.8 - 2.3) | NS |  | 1.2 | (0.5 - 2.6) | NS |  | 0.8 | (0.3 - 2.2) | NS |
|  | Other psychotic conditions | Yes | 1.0 | (0.5 - 1.9) | NS |  | 2.1 | (0.6 - 7.5) | NS |  | 2.1 | (0.3 - 13.5) | NS |

^a^ Significance: P-value ≤0.05 ‡, P-value >0.05 NS.

^b^ Data not shown due to extreme confidence limits.

HR is >1 if a variable is associated with subsequent self-harm, =1 if not associated, and <1 if inversely associated.

| (a) |  | (b) |  |
| --- | --- | --- | --- |
|  | 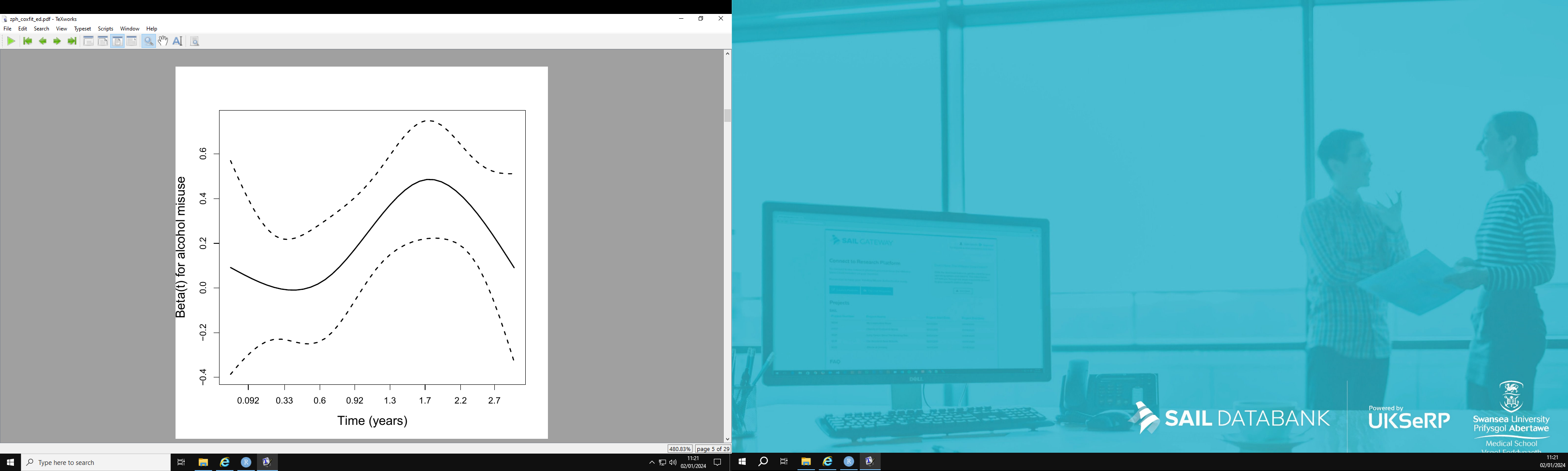 |  | 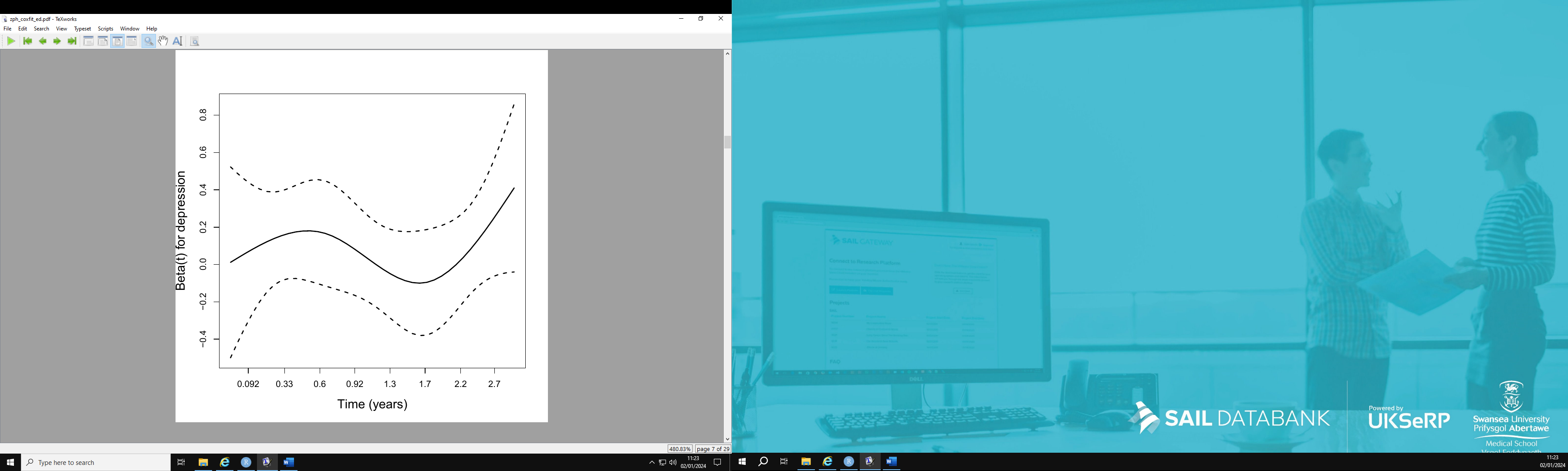 |
| (c) |  |  |  |
|  | 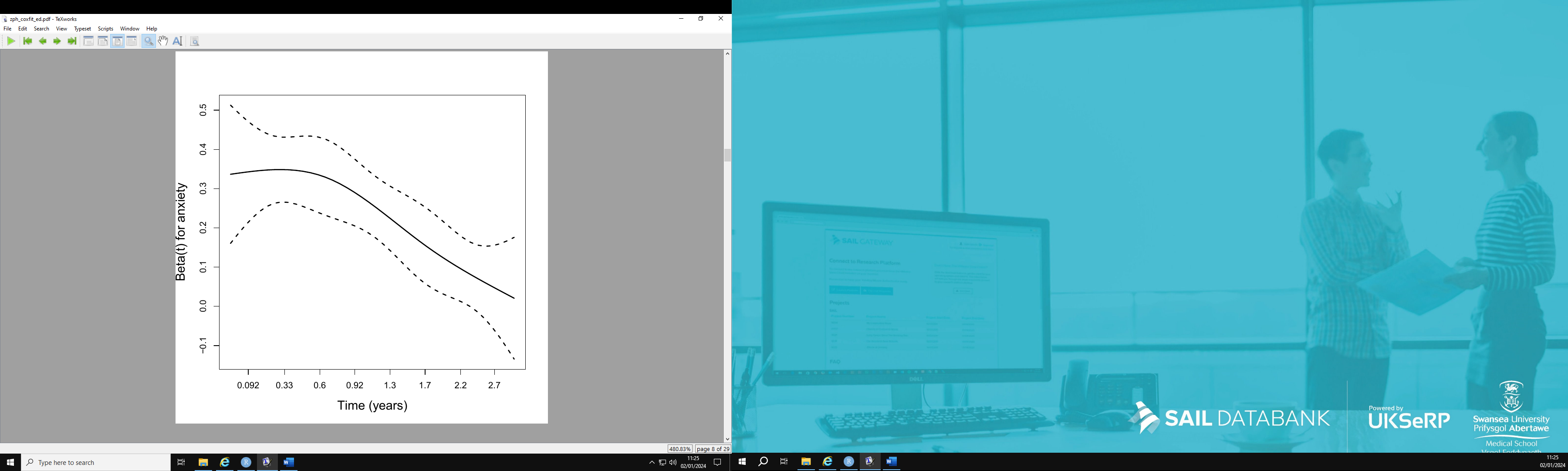 |  |  |

**Supplementary F1**. Spline fit of time varying estimates of the log of the hazard ratio for alcohol use (a), depression(b) and anxiety (c) to determine cutoff times for time-stratified Cox regressions.

**Supplementary F2.** Flow diagram of prisoner and non-prisoner cohort selection. For survival analyses, imprisonment/index dates for the prisoner and non-prisoner cohorts were restricted to those from 2013 and after. These individuals had pre-imprisonment/counterfactual index dates defined three years before the respective imprisonment/index dates. These individuals were then included if they met Welsh residency and GP data criteria at the pre-imprisonment/counterfactual index dates.

| 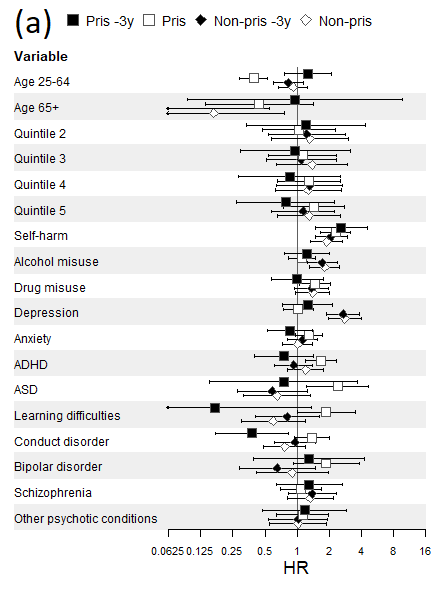 | 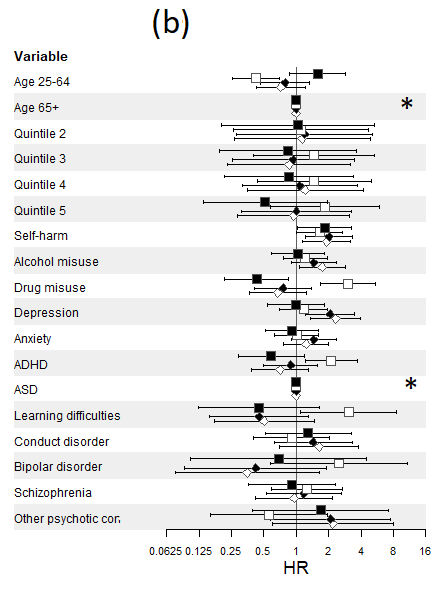 | 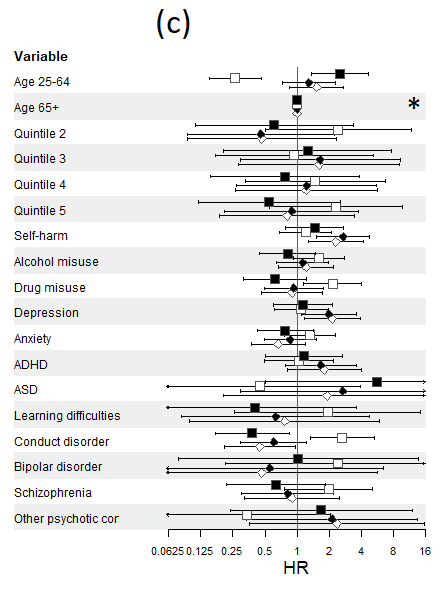 |
| --- | --- | --- |

**Supplementary F3.** Associations between previous self-harm/mental health conditions and subsequent self-harm in prisoners and non-prisoners. Hazard ratios come from a Cox regression using four groups: ‘prisoners’ and ‘non-prisoners’ starting at imprisonment/index date, and ‘prisoners - 3 years’ and ‘non-prisoners - 3 years’ starting at pre-imprisonment/counterfactual index date. Separate analyses were done for the three time periods (a) 0 to 0.6 years, (b) 0.6 to 1.6 years and (c) 1.6 to 3 years. Asterisks show where extreme confidence limits could not be displayed. HR is >1 if a variable is associated with subsequent self-harm, =1 if is not associated, and <1 if it is inversely associated.


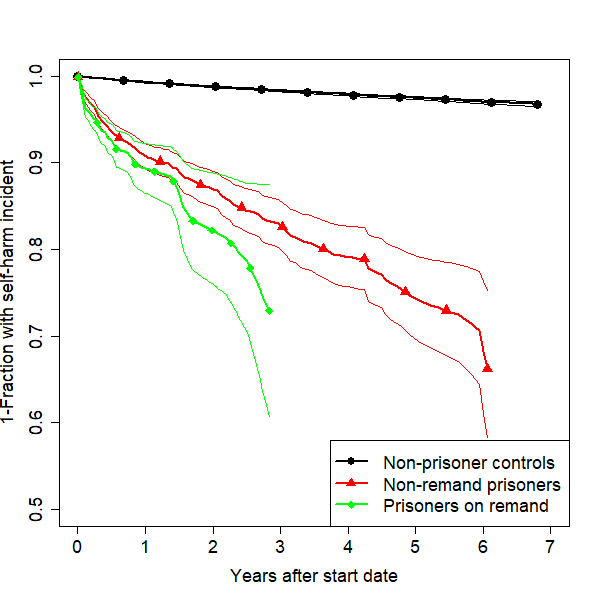


**Supplementary F4.** Kaplan-Meier plot showing 1- the fraction with self-harm incident whilst in prison or after the index date for a non-prisoner. Curves with confidence intervals are shown for non-prisoner, sentenced prisoners and remand prisoners. A steeper curve indicates a faster accumulation of subsequent self-harm events. A lower end-point indicates higher subsequent self-harm risk.
